# Phosphatidylinositol synthesis, its selective salvage, and inter-regulation of anionic phospholipids in *Toxoplasma gondii*

**DOI:** 10.1038/s42003-020-01480-5

**Published:** 2020-12-10

**Authors:** Bingjian Ren, Pengfei Kong, Fatima Hedar, Jos F. Brouwers, Nishith Gupta

**Affiliations:** 1 Department of Molecular Parasitology, Faculty of Life Sciences, Humboldt University, Berlin, Germany; 2 Center for Molecular Medicine, University Medical Center, Utrecht, The Netherlands; 3 Department of Biological Sciences, Birla Institute of Technology and Science Pilani (BITS-P), Hyderabad, India

**Keywords:** Parasite biology, Lipidomics

## Abstract

Phosphatidylinositol (PtdIns) serves as an integral component of eukaryotic membranes; however, its biosynthesis in apicomplexan parasites remains poorly understood. Here we show that *Toxoplasma gondii*—a common intracellular pathogen of humans and animals—can import and co-utilize *myo*-inositol with the endogenous CDP-diacylglycerol to synthesize PtdIns. Equally, the parasite harbors a functional PtdIns synthase (PIS) containing a catalytically-vital CDP-diacylglycerol phosphotransferase motif in the Golgi apparatus. Auxin-induced depletion of PIS abrogated the lytic cycle of *T. gondii* in human cells due to defects in cell division, gliding motility, invasion, and egress. Isotope labeling of the PIS mutant in conjunction with lipidomics demonstrated de novo synthesis of specific PtdIns species, while revealing the salvage of other lipid species from the host cell. Not least, the mutant showed decline in phosphatidylthreonine, and elevation of selected phosphatidylserine and phosphatidylglycerol species, indicating a rerouting of CDP-diacylglycerol and homeostatic inter-regulation of anionic phospholipids upon knockdown of PIS. In conclusion, strategic allocation of own and host-derived PtdIns species to gratify its metabolic demand features as a notable adaptive trait of *T. gondii*. Conceivably, the dependence of *T. gondii* on de novo lipid synthesis and scavenging can be exploited to develop new anti-infectives.

## Abstract (web summary)

Ren et al. investigated how the obligate intracellular pathogen *Toxoplasma gondii* satisfies its requirement of phosphatidylinositol (PtdIns). They show that this parasite can synthesize certain PtdIns species de novo and salvages others from human host cells. Endogenous production of PtdIns in the Golgi complex is critical for the acute infection, and its loss perturbs homeostasis of selected anionic phospholipids.

## Introduction

Asexual reproduction of apicomplexan parasites in respective host cells involves successive rounds of lytic cycle leading to acute infection. A corresponding biogenesis of organellar membranes is imperative to sustain intracellular proliferation of these parasites. Previous research on *Toxoplasma*, *Plasmodium,* and *Eimeria* has revealed the occurrence of lipid biosynthesis networks in these parasites, occasionally involving a balance of endogenous synthesis and salvage of lipids from sheltering host cells^1–3^. Our own work focusing on phospholipids has demonstrated the expression and synthesis of generic, as well as exclusive lipids in *T. gondii* and *E. falciformis*^3–9^. In context of this report, we showed that the acute stage of *T. gondii* can synthesize several major classes of phospholipids, namely phosphatidylcholine (PtdCho), phosphatidylethanolamine (PtdEtn), phosphatidylthreonine (PtdThr), and phosphatidylserine (PtdSer). Among these, PtdThr is an exclusive anionic lipid occurring only in coccidian parasites, which regulates calcium homeostasis in *T. gondii*^10,11^. PtdThr, PtdCho, and PtdSer are made in the endoplasmic reticulum (ER), whereas PtdEtn is produced at multiple locations including the ER, mitochondrion and parasitophorous vacuole (PV)^3–9^. PtdIns is yet-another primary class/family of membrane phospholipids in *T. gondii*; however, its biosynthesis and importance in the parasite have not yet been examined.

In parasitic protists, PtdIns itself and its derivative lipids are known to facilitate a repertoire of functions. Especially, metabolism of PtdIns-derived second messengers (phosphoinositides) has drawn notable attention due to their crucial roles in parasite pathogenesis and potential as the target for drugs, vaccines or diagnosis^12–20^. They are considered essential cellular mediators involved in apicoplast homeostasis (*Toxoplasma*)^21,22^, protein export (*Plasmodium*)^23,24^, motility and egress (*Plasmodium*)^25,26^, endocytosis (*Trypanosoma*)^27,28^, autophagy (*Plasmodium*, *Trypanosoma*)^29,30^, and gametogenesis (*Plasmodium*)^31^. Another vital class of metabolites is glycosylphosphatidylinositol (GPI), which serves as a membrane anchor to glycoproteins. Several GPI-anchored surface proteins have been implicated in modulation of the host’s immune response^32–35^ and parasite survival^36,37^. A multifarious requirement of PtdIns species and metabolites derived thereof has necessitated understanding the mechanism and regulation of PtdIns synthesis in protozoan parasites.

Synthesis of PtdIns is catalyzed by PtdIns synthase (PIS), which co-utilizes CDP-diacylglycerol (CDP-DAG) and *myo*-inositol as precursors^38^. Thus far only one protozoan PIS, isolated from the kinetoplastid parasite, *T. brucei*, has been studied in substantial details. *Tb*PIS localizes in the ER and Golgi network of the blood stages of *T. brucei*^39^, generating two distinct PtdIns pools. In the ER, de novo-synthesized *myo*-inositol is utilized to produce PtdIns, which subsequently supports GPI synthesis^40,41^. On the other hand, PtdIns production in the Golgi complex employs exogenous *myo*-inositol, and this lipid pool is used to synthesize inositol phosphorylceramide^42,43^. Among intracellular parasites, the presence of PIS enzymes has been reported in *Plasmodium*, *Toxoplasma,* and *Eimeria*^3,44–46^, although their contribution to the making of different PtdIns species and their physiological relevance have not been investigated. In addition, it is unclear how these parasites meet a balance between the synthesis of PtdIns and its import from host cells. In this work, we characterized PtdIns synthase of *T. gondii* and demonstrated a vital role of de novo PtdIns synthesis along with the salvage of designated host-derived lipid species by the parasite.

## Results

### *T. gondii* can synthesize PtdIns by co-utilizing *myo*-inositol and CDP-DAG

**Fig. 1:**
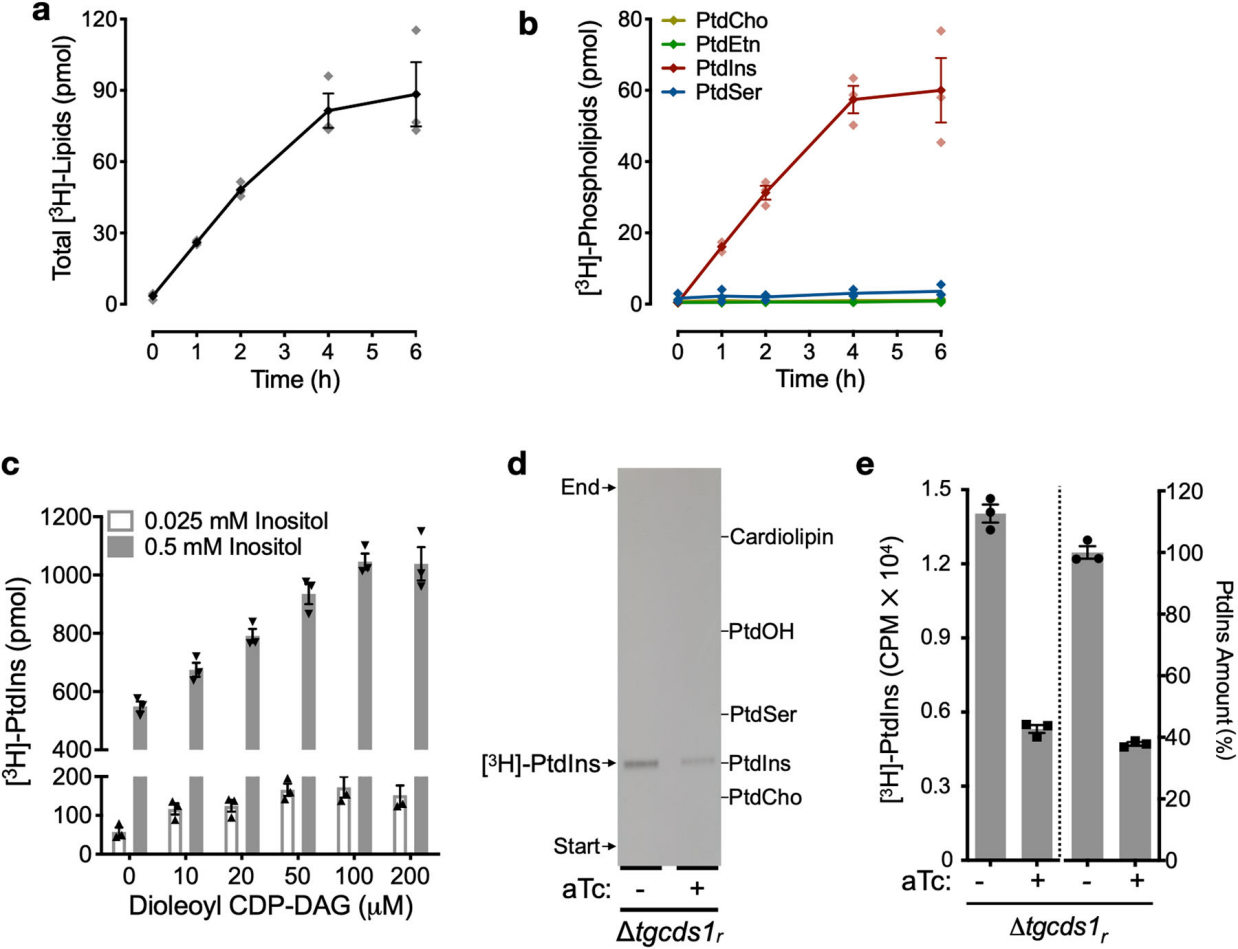
Extracellular tachyzoites of *Toxoplasma gondii* can generate PtdIns by co-utilizing *myo*-inositol and CDP-diacylglycerol. **a–c** Radiolabeling of purified tachyzoites of the RH strain with *myo*-inositol. Parasites (10^8^/reaction) were incubated with [^3^H]-*myo*-inositol (10 μCi) in the labeling medium for indicated periods, immediately followed by lipid extraction. Incorporation of radiotracer was quantified in total lipids (**a**) and in the major glycerophospholipid classes resolved by thin-layer chromatography (**b**). A conversion of CPM counts scored by liquid scintillation counter into absolute amounts was achieved using the formula (X CPM = Y pmol) derived from the reference counts in the substrate preparation (10 μCi, 0.025 mM). **c** shows the same assay as depicted in **a**, **b** except for that CDP-DAG and *myo*-inositol were included at specified concentrations (duration, 4 h). **d** Autoradiogram showing TLC-resolved lipids from radiolabeled parasites of the Δ*tgcds1*_*r*_ strain under *on* (-aTc) and *off* (+aTc) conditions. The Δ*tgcds1*_*r*_ strain was precultured in aTc (1 μM) for two passages (96 h) prior to radiolabeling assay. Fresh parasites (5 × 10^7^/reaction) were incubated with [^3^H]-*myo*-inositol (10 μCi, 0.5 mM) for 4 h. Lipids were separated by TLC and visualized by X-ray detection (–80 °C, 40 h). **e** Quantification of PtdIns labeling and amount in the Δ*tgcds1*_*r*_ mutant. The left *Y-axis* shows the mean scintillation counts in TLC-scraped bands from **d**. The right *Y*-axis illustrates the steady-state amount of PtdIns in the mutant precultured in the absence or presence of aTc. The data in **a**–**c** and **e** represent the mean with S.E. from 3 independent experiments.

To investigate whether the parasite can import and utilize exogenous inositol for PtdIns synthesis, we incubated fresh extracellular tachyzoites with [^3^H]-*myo*-inositol. Parasites exhibited a time-dependent incorporation of *myo*-inositol into lipids, which was nearly linear during the first 4 h of incubation and then gradually decelerated afterwards (Fig. 1a). Separation of total lipids from radiolabeled tachyzoites by thin-layer chromatography (TLC) revealed that among key phospholipids, only PtdIns was labeled with [^3^H]-*myo*-inositol (Fig. 1b). In similar assays using a saturating amount of *myo*-inositol (0.5 mM), we observed a significant surge in PtdIns synthesis (Fig. 1c), confirming a substrate-dependence of the process. Likewise, the addition of CDP-DAG as a co-substrate further stimulated the synthesis of PtdIns, though modestly, under low as well as high *myo-*inositol conditions. We also estimated the maximal rates of PtdIns synthesis by extracellular tachyzoites of the RH strain, which was about 0.25 nmol/h for an aliquot of 10^8^ cells. The rate of lipid synthesis is circa 25% of the amount needed for a cell doubling based upon a content of 8 nmol PtdIns/10^8^ cells. The synthetic capacity of tachyzoites in extracellular milieu thus appears to be much lower than the lipid demand imposed by intracellular proliferation.

Notably, increased supply of CDP-DAG up to 0.2 mM led to accentuated biosynthesis of PtdIns, albeit the dose-dependence of lipid synthesis on CDP-DAG was not as proportionate as observed with *myo*-inositol (Fig. 1c). In other words, a 20-fold increase in *myo*-inositol (from 0.025 to 0.5 mM) caused a 10x higher lipid synthesis, whereas a 20-fold increase in exogenous CDP-DAG (from 10 to 200 µM) yielded only a 2-fold additional labeling. We, therefore, tested the notion of whether the parasite may depend on CDP-DAG made de novo (Fig. 1d–e). We performed [^3^H]-*myo*-inositol labeling of an anhydrotetracycline (aTc)-repressible mutant of CDP-DAG synthase 1 located in the endoplasmic reticulum (Δ*tgcds1*_*r*_)^7^. Indeed, knockdown of *Tg*CDS1 caused a strong reduction in [^3^H]-*myo*-inositol incorporation into PtdIns (Fig. 1d–e). Consistently, the steady-state amount of PtdIns was also reduced to the same extent in the *off-state* Δ*tgcds1*_*r*_ mutant. Taken together, the data show that tachyzoites are competent in making PtdIns by co-utilizing exogenously supplied *myo*-inositol and endogenously-produced CDP-DAG.

### *T. gondii* harbors a functional PtdIns synthase in the Golgi complex

**Fig. 2:**
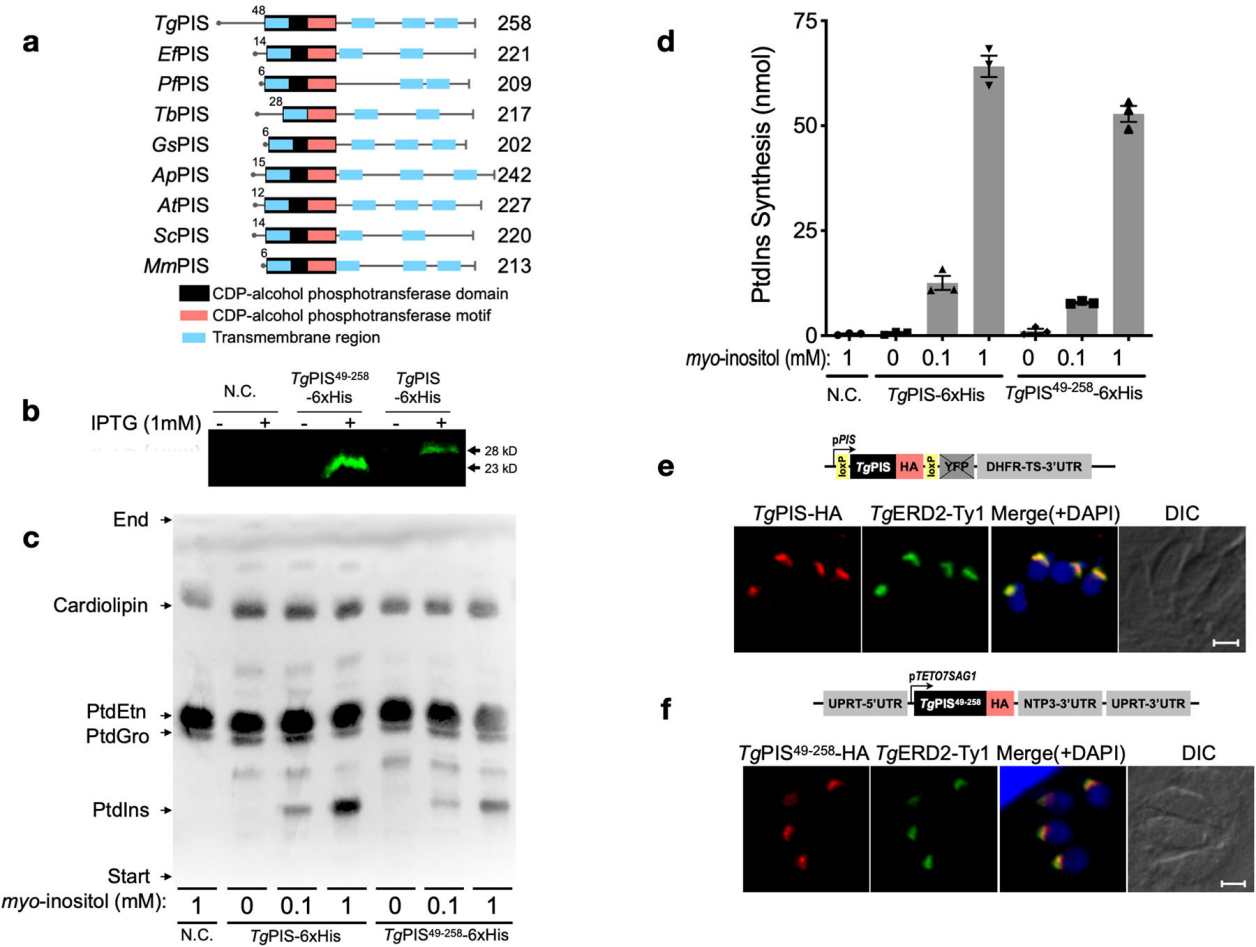
Tachyzoites express a functional PtdIns synthase in the Golgi complex. **a** The primary structures of *Tg*PIS and selected orthologs from different organisms. CDP-alcohol phosphotransferase domains and transmembrane regions were predicted by Simple Modular Architecture Research Tool (SMART) and transmembrane Hidden Markov Model (HMM). Numerals indicate the size of the N-termini and entire open reading frames in amino acids. Other relevant sequence information such as, GenBank IDs are shown in Supplementary Table 1. *Ef*, *Eimeria falciformis*; *Pf*, *Plasmodium falciparum*; *Tb*, *Trypanosoma brucei*; *Gs*, *Galdieria sulphuraria*; *Ap*, *Auxenochlorella protothecoides*; *At*, *Arabidopsis thaliana*; *Sc*, *Saccharomyces cerevisiae*; *Mm*, *Mus musculus*. **b** Immunoblot of *E. coli* M15/pREP4 strains harboring *pQE60* (N.C., negative control), *pQE60-TgPIS-*6xHis or *pQE60-TgPIS*^49–258^*-6xHis* plasmids. Induction was performed by IPTG as reported in “Methods”. The total bacterial extract was resolved by 12% SDS-PAGE, blotted and probed with the mouse anti-His antibody (1:3000). **c** TLC of lipids isolated from *E. coli* samples as shown in **b**. Expression was induced by 1 mM IPTG, and cultures were incubated with specified amounts of *myo*-inositol. Lipids were visualized under the UV light after spraying 8-anilino-1-naphthalenesulfonic acid. The unlabeled bands in samples on the TLC plate represent unknown lipids, some of which appear upon expression of PIS isoforms regardless of *myo*-inositol substrate in medium. **d** Lipid-phosphorous quantification of bands corresponding to PtdIns in **c**. Silica scraping containing the individual lipid bands were subjected to lipid-phosphorus measurements along with defined standards. Graph shows the mean values with S.E. from 3 different assays. **e**, **f** Immunofluorescence images of the transgenic tachyzoites expressing either *Tg*PIS-HA or *Tg*PIS^49–258^-HA. The strain expressing *Tg*PIS-HA under the control of its native promoter and *Tg*DHFR-TS-3′UTR was constructed as described in Fig. 3a. The truncated isoform lacking the extended N-terminal sequence (1–48 residues, **a**) was regulated by p*TETO7SAG1* promoter and *Tg*NTP3–3′UTR. The *Tg*PIS^49–258^-HA expression cassette was inserted at the *UPRT* locus by negative selection using 5-fluorodeoxyuridine. Transgenic parasites expressing the either variants were transfected with a vector expressing *Tg*ERD2-Ty1 (regulated by *TgGRA1* elements) for co-localization study. Immunostaining was performed 24 h post-infection using α-HA/Alexa594 (red) and α-Ty1/Alexa488 (green) antibodies. Nuclei were stained with DAPI (blue). Scale bars, 2 μ; *DIC*, differential interference contrast.

We next focused on establishing the genetic basis of PtdIns synthesis in *T. gondii*. Previous work has reported the occurrence of a “tachyzoite-specific” isoform that can functionally complement a PIS mutant of *Saccharomyces cerevisiae*^46^. This protein (ToxoDB, TGGT1_207710; NCBI GenBank, KX017549) comprises 258 residues with four transmembrane helices and a CDP-alcohol phosphotransferase domain with D*X*_2_DG*X*_2_AR*X*_8/9_G*X*_3_D*X*_3_D motif (Fig. 2a). Besides several residues conserved in CDP-alcohol phosphotransferase-type enzymes (Supplementary Table 1), we identified glutamine (Q103) and arginine (R117), present only in the PIS sequences (Supplementary Figure 1a, Supplementary Table 1). *Tg*PIS-6xHis was expressed in *Escherichia coli*, which lacks the PtdIns synthase activity, and thus well suited for functional analysis of the PIS proteins. Successful IPTG-inducible expression of the full-length recombinant protein was confirmed by Western blot analysis (28-kDa band, Fig. 2b). The catalytic activity was assessed by thin-layer chromatography in conjunction with the quantification of phosphorus in TLC-resolved lipid bands (Fig. 2c–d). As expected, the *E. coli* strain harboring the empty vector (negative control, N.C.) did not show any PtdIns synthesis, whereas the expression of *Tg*PIS-6xHis produced PtdIns in a *myo*-inositol-dependent manner. The site-mutations or deletion of most conserved residues abolished the catalysis except for Q103G mutation, which could still produce PtdIns, albeit with a notably reduced efficiency (Supplementary Figure 1a–c), which endorsed the functional importance of the predicted signature residues.

In our preceding work^7^, we have reported localization of ectopically overexpressed *Tg*PIS in the Golgi network. Herein, we expressed *Tg*PIS-HA under the control of its endogenous promoter and found it exclusively in the Golgi body, as determined by co-localization with a known organelle marker *Tg*ERD2^47^ (Fig. 2e). In further work, we tested the relevance of a prolonged N-terminal extension in *Tg*PIS (Fig. 2a) by ectopically expressing a mutant lacking the specified extension (*Tg*PIS^49–258^-HA). A single copy of the PIS expression cassette directed by the p*TETO7SAG1* regulatory elements was inserted at the uracil phosphoribosyltransferase (UPRT) locus by double homologous recombination (Fig. 2f). *Tg*PIS^49–258^-HA mutant localized in the Golgi complex, as judged by its co-staining with *Tg*ERD2-Ty1. We also performed expression of *Tg*PIS^49–258^-6xHis in *E. coli* (23-kDa band, Fig. 2b), but observed only a minor reduction in PtdIns synthesis by the truncated mutant (Fig. 2c–d). Based on all these results, we conclude that *Tg*PIS encodes a functionally-active enzyme, which localizes in the parasiteʼs Golgi apparatus, and its extended N-terminus is dispensable for the subcellular localization and catalytic activity.

### PtdIns synthase is essential for the lytic cycle of *T. gondii*

**Fig. 3:**
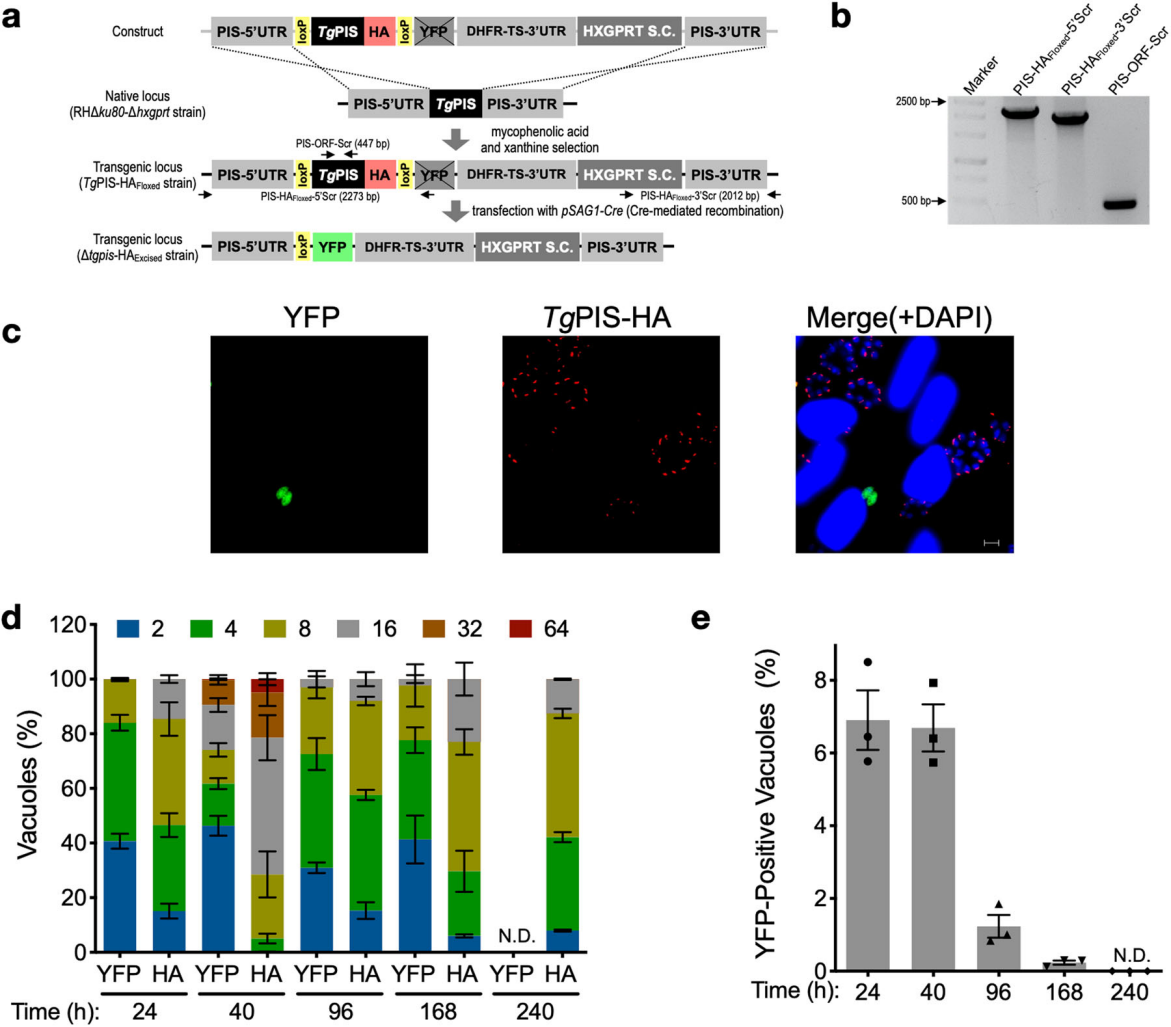
Cre-mediated excision of *Tg*PIS abrogates the parasite growth. **a** Scheme illustrating the gene deletion strategy. In the first step, the *TgPIS* gene in the RHΔ*ku80*Δ*hxgprt* strain was replaced by a cassette comprising the loxP-flanked (floxed) HA-tagged open reading frame of *Tg*PIS (*Tg*PIS-HA), YFP sequence and HXGPRT selection cassette (S.C.), creating the *Tg*PIS-HA_Floxed_ strain. The second step involved a transient transfection of the *Tg*PIS-HA_Floxed_ strain with the *pSAG1-Cre* construct to induce Cre-mediated recombination, which excised *Tg*PIS-HA and repositioned YFP sequence in the eventual Δ*tgpis*-HA_Excised_ mutant. Note that YFP in not expressed in the *Tg*PIS-HA_Floxed_ strain, but Cre-mediated excision of *Tg*PIS-HA brings it in the proximity of the PIS promoter, enabling its expression in the Δ*tgpis*-HA_Excised_ strain. **b** Genomic PCR of the *Tg*PIS-HA_Floxed_ strain confirming the 5′- and 3′-crossovers and insertion of *Tg*PIS-HA. Primers used for PCR-screening at the *TgPIS* locus are marked as arrows in **a**. **c** Immunofluorescence images of *Tg*PIS-HA (red) and YFP (green) in the Δ*tgpis*-HA_Excised_ strain 24 h post-transfection with the *pSAG1-Cre* plasmid. Nuclei were stained with DAPI (blue). Scale bars: 5 μ. **d** Quantification of the parasitophorous vacuoles with tachyzoites expressing YFP or HA signal at different times after *Cre* expression. The number of tachyzoites/vacuoles was scored in 400–500 vacuoles for each strain, and different colors signify their variable numbers. **e** The proportion of parasitophorous vacuoles with YFP signal in the Δ*tgpis*-HA_Excised_ mutant. Data in **d**, **e** show mean values with S.E. from 3 independent assays (N.D., not detectable).

We attempted to delete the *TgPIS* gene by double homologous recombination in tachyzoites of *T. gondii*. However, the locus was refractory to deletion, suggesting a vital requisite of this enzyme during the lytic cycle. To confirm our premise, we implemented the Cre-mediated gene-swap strategy^48^. In this regard, the *TgPIS* locus was first replaced by a cassette comprising the loxP-flanked (floxed) open reading frame of *Tg*PIS-HA, followed by yellow fluorescence protein (YFP), *Tg*DHFR-TS-3′UTR, and *Tg*HXGPRT selection cassette (Fig. 3a). The method enabled us to get a viable strain (*Tg*PIS-HA_Floxed_), as confirmed by genomic PCR of clonal transgenic parasites (Fig. 3b). Transfection of the *Tg*PIS-HA_Floxed_ strain with the *pSAG1-Cre* construct expressing Cre recombinase caused the excision of floxed *Tg*PIS-HA, and repositioned YFP in proximity of the *Tg*PIS promoter that resulted in fluorescing Δ*tgpis*-HA_Excised_ mutant (Fig. 3c). The mutant could not be drug-selected because it was not feasible to generate a clonal knockout strain. Nevertheless, it allowed us to investigate the effect of genetic ablation on the parasite growth by scoring the progression of the YFP-positive Δ*tgpis*-HA_Excised_ strain up to 10 days of transfection (Fig. 3d–e). Indeed, parasites without *Tg*PIS-HA signal but expressing YFP had much smaller vacuoles, and vice versa, indicating a replication defect. The YFP-positive vacuoles disappeared from mixed cultures within 7–10 days (Fig. 3e), disclosing an indispensable role of *Tg*PIS during the lytic cycle.

### Auxin-induced degradation of *Tg*PIS function is detrimental to tachyzoites

**Fig. 4:**
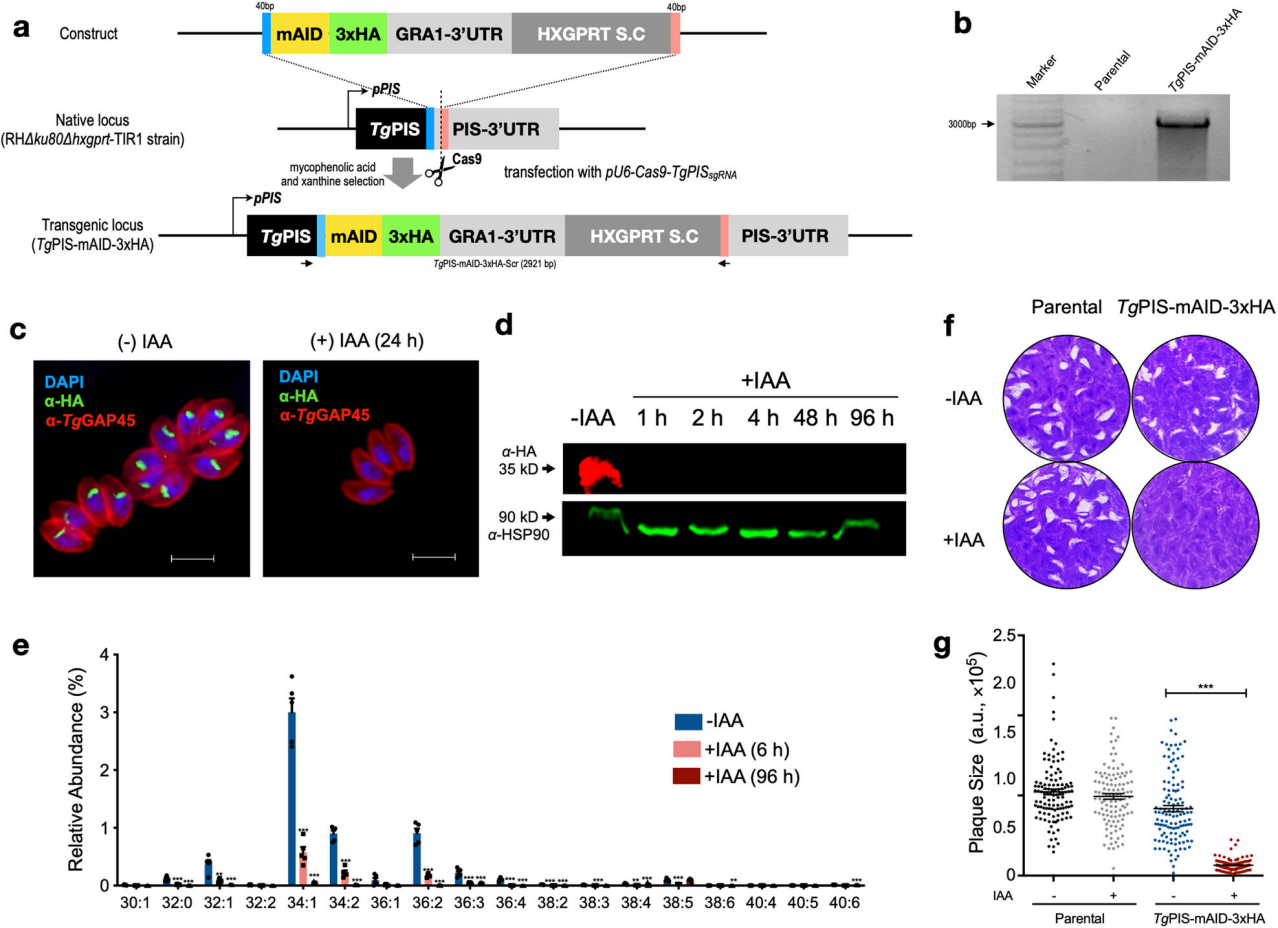
Auxin-induced ablation of PtdIns synthesis blights the lytic cycle of tachyzoites. **a** Scheme showing the Cas9-assisted 3′-genomic tagging of *Tg*PIS with a minimal auxin-inducible degron (mAID) and 3xHA epitope. Indicated amplicon (mAID-3xHA-GRA1–3′UTR-HXGPRT) flanked with short (40 bp) homology arms was co-transfected with the *pU6-Cas9-TgPIS*_*sgRNA*_ vector (expressing Cas9 and *single guide* RNA) into the RHΔ*ku80*Δ*hxgprt*-TIR1 strain, which was then drug-selected for the HXGPRT selection cassette (S.C.). The ensuing *Tg*PIS-mAID-3xHA strain expressed C-terminally epitope-tagged *Tg*PIS, and enabled its conditional knockdown by auxin treatment. **b** PCR confirming the event of 3′-insertional tagging in the *Tg*PIS-mAID-3xHA strain. Primers used for the genomic screening at the *TgPIS* locus are marked as arrows in **a**. **c** Images showing a dependence of *Tg*PIS expression on auxin in the *Tg*PIS-mAID-3xHA mutant. Parasites were cultured with 500 µM IAA for 24 h, and stained with α-HA and α-*Tg*GAP45 antibodies to visualize PIS (Golgi, green) and GAP45 (inner membrane complex, red). DAPI highlights the parasite and host-cell nuclei. **d** Immunoblot of the *Tg*PIS-mAID-3xHA strain treated with 500 μM IAA for varying periods. The fusion protein was stained with α-HA antibody, and *Tg*HSP90 served as the loading control. **e** [^13^C]-PtdIns synthesis in the extracellular tachyzoites of the *Tg*PIS-mAID-3xHA mutant cultured without or with auxin, as shown. Fresh tachyzoites (1 × 10^7^) treated with 500 μM IAA or carrier solvent (0.1% ethanol, -IAA) were labeled with 0.5 mM [^13^C]-*myo*-inositol (6 h, 37 °C), followed by lipidomic analysis (*n* = 5 assays). **f**, **g** Plaque assays revealing growth fitness of the *Tg*PIS-mAID-3xHA mutant and parental strains. The crystal violet-stained images (**f**) reveal plaques formed by successive lytic cycles of tachyzoites in the absence or presence of IAA (500 μM). The plaque area, depicted as arbitrary units (a. u.) in **g**, was measured by ImageJ. Over 100 plaques for each strain from 3 independent assays were scored. Data in **e** and **g** show the mean ± S.E (*p ≤ 0.05; **p ≤ 0.01; ***p ≤ 0.001).

A viable *Tg*PIS deletion mutant could not be generated, hence we implemented the conditional mutagenesis by tagging the enzyme with an auxin-inducible degradation (AID) domain for translational control of the protein stability^49^. In this regard, a CRISPR/Cas9 construct expressing a *single guide* RNA targeted to the 3′UTR of the *TgPIS* gene (*pU6-Cas9-TgPIS*_*sgRNA*_) was co-transfected with a PCR-amplicon containing the 5′- and 3′-homology arms, minimal AID (mAID) motif, 3xHA tag, GRA1–3′UTR and HXGPRT selection marker to permit crossover-mediated epitope-tagging of the native locus by positive selection (Fig. 4a). Successful mAID-3xHA-tagging of *TgPIS* was confirmed by recombination-specific PCR (Fig. 4b), its subcellular localization in the Golgi apparatus (Fig. 4c), and immunoblot analysis revealing the expected 35-kDa band (Fig. 4d). As reckoned, the HA signal was not detectable after auxin exposure (Fig. 4c–d).

The AID method resulted in a strain that allowed us to undertake biochemical and phenotypic analyses. [^13^C]-*myo*-inositol labeling of the auxin-treated extracellular tachyzoites caused a significant reduction in the synthesis of most detectable PtdIns species, confirming a functional ablation of PIS and a role of enzyme in de novo synthesis of several lipid species (Fig. 4e). Although a depletion of enzyme activity was evident within 6 h of auxin treatment, it was more pronounced (nearly complete) after 96 h. As anticipated, none other [^13^C]-labeled phospholipid was detected in the mutant irrespective of the auxin exposure. The content of major phospholipids except for PtdIns, PtdGro, and PtdThr were unaltered (Supplementary Figure 2, also see below). In plaque assays, we observed a severely impaired parasite growth following the hormone-mediated depletion of PIS (Fig. 4f–g). The plaque size was reduced to less than 10% when *Tg*PIS-mAID-3xHA mutant was treated with auxin. Not least, the parasite ceased to grow after 3–4 passages in routine cultures, once more revealing a critical role of PtdIns synthesis for the lytic cycle.

### Auxin-mediated depletion of PIS impairs all major events during the lytic cycle

**Fig. 5:**
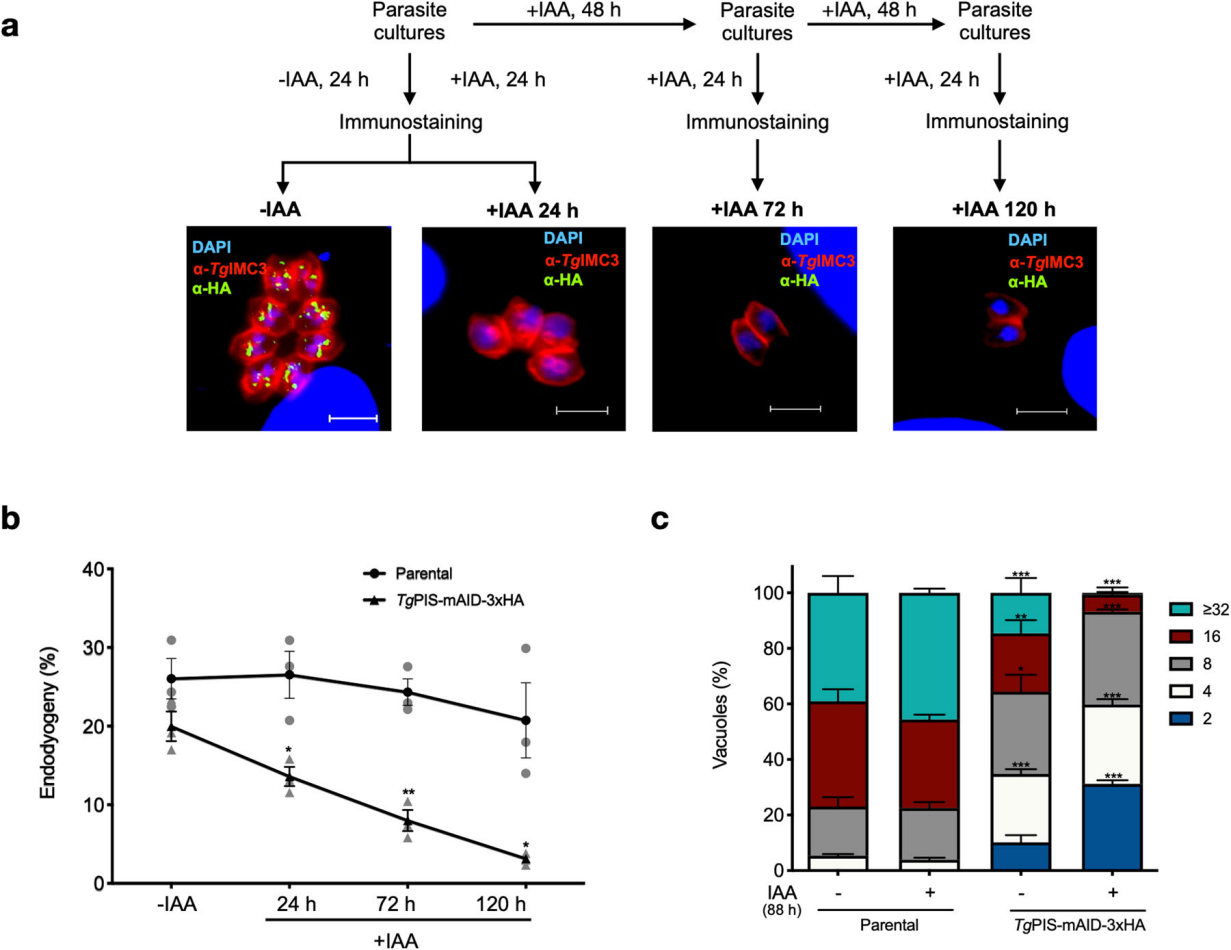
Depletion of *Tg*PIS impairs the cell division in tachyzoites. **a** Immunostaining of the budding daughter cells (endodyogeny) in the *Tg*PIS-mAID-3xHA strain precultured without or with auxin. Intracellularly-developing tachyzoites were treated with 500 μM IAA or with 0.1% ethanol (-IAA), as schematized. Samples were stained with α-HA (green) to monitor the downregulation of *Tg*PIS, and with α-*Tg*IMC3 (red) to visualize the endogeny. Shown are the representative images of *Tg*PIS-mAID-3xHA mutant. **b** Quantification of tachyzoites harboring the progeny. A total of 400–500 vacuoles with IMC3-positive daughter cells were scored for each condition (*n* = 3 assays). **c** Proliferation rate of the *Tg*PIS-mAID-3xHA mutant with respect to its parental strain. Parasites were precultured with 500 µM IAA ( + IAA) or 0.1% ethanol (-IAA) for 48 h and subjected to the replication assay (40 h infection). Graphs show the mean percentage of vacuoles containing specified number of parasites. A total of 400–500 vacuoles for each condition were scored (*n* = 3 assays, mean ± S.E; *p ≤ 0.05; **p ≤ 0.01; ***p ≤ 0.001).

The auxin-regulated *Tg*PIS-mAID-3xHA mutant also enabled phenotyping of the lytic cycle events such as, replication, egress, invasion, and gliding motility (Fig. 5, Supplementary Figure 3). Assuming a standard role of PtdIns in membrane biogenesis, we first set up immunofluorescent assays to monitor the formation of daughter cells (endodyogeny) by staining with the anti-IMC3 antibody^50^ before and after auxin exposure (Fig. 5a). Compared to the parental strain, a minor impairment in the cell division of the PIS mutant was observed even under the control condition (Fig. 5b) that can be attributed to the mAID-3xHA tagging and GRA1–3′UTR. More importantly, unlike the parental strain, the proportion of budding cells declined gradually in the PIS mutant upon prolonged exposure to the hormone (24–120 h). In accord with these and aforesaid findings using the Δ*tgpis*-HA_Excised_ mutant (Fig. 3c–d), we recorded a significant defect in the intracellular replication of the auxin-treated *Tg*PIS-mAID-3xHA strain (Fig. 5c). Yet again, as shown above, a modest decline in the proliferation of the mutant was apparent in the absence of auxin.

*Tg*PIS-mAID-3xHA mutant also exhibited egress defect in normal cultures (-IAA), which was radically accentuated upon inclusion of auxin (Supplementary Figure 3a). To test whether curtailed egress may be caused by slow replication of the mutant, we examined induced egress in response to activation of cGMP signaling by zaprinast, a phosphodiesterase inhibitor^51,52^. Indeed, we recorded a marked recovery in egress of the auxin-treated mutant but the impairment was still evident (Supplementary Figure 3b). Likewise, we noted a reduction in invasion efficiency of the mutant in auxin-supplemented cultures, albeit only after 96 h (Supplementary Figure 3c). Last but not least, our appraisal of the parasite motility demonstrated a modest but significant reduction in the motile fraction and trail length of the PIS mutant after auxin exposure (Supplementary Figure 3d–e). Collectively, our results show a multifaceted impact of PtdIns synthase depletion on the lytic cycle of tachyzoites.

### Knockdown of PtdIns synthase disrupts homeostasis of major anionic lipids

To gain insight into observed phenotypes, auxin-regulated conditional mutant of *Tg*PIS was subjected to lipidomic analysis. In this regard, we first performed the parasite yield assay, as reported earlier^53^, to determine a suitable time point for the sample collection (Supplementary Figure 4a). Parasitized cultures of the mutant and parental strains with a defined multiplicity of infection were incubated in the absence or presence of auxin and the parasite yield was calculated after consecutive passages. As expected, the growth rate of the parental strain remained unaltered irrespective of hormone treatment. In contrast, the yield of auxin-exposed mutant cultures declined gradually during two serial passages. In other assays, we confirmed the viability and infectivity of these parasites, both of which were normal after 96 h treatment when compared to untreated samples. Besides, we immunostained major organelles, namely plasma membrane, IMC, apicoplast, mitochondrion, and ER (Supplementary Figure 4b). No apparent difference was observed in hormone-treated *vs*. untreated control samples for any of these organelles. All indicated marker proteins including the GPI-anchored SAG1 and SAG2 were targeted and expressed with no marked anomaly.

**Fig. 6:**
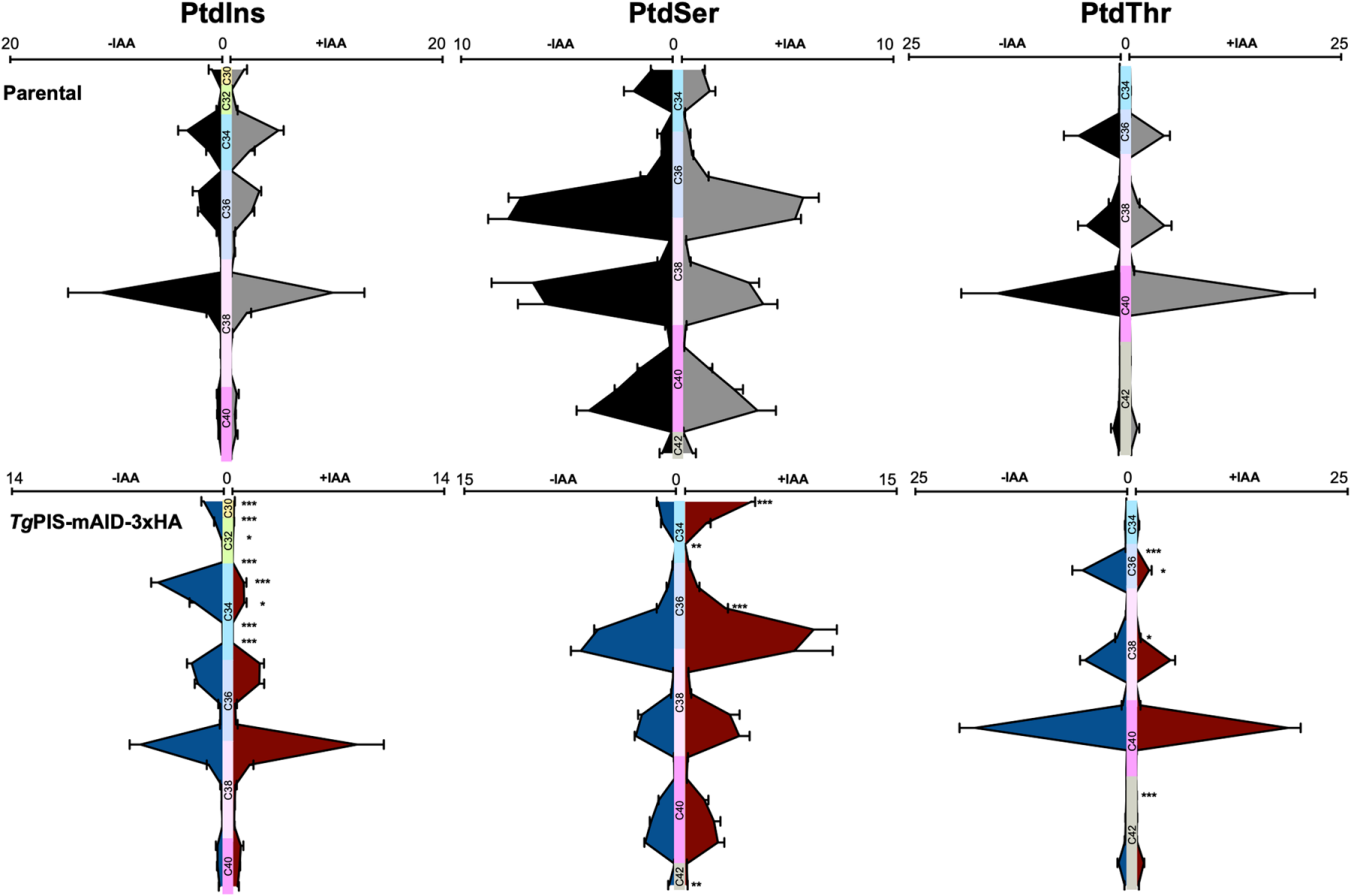
Auxin-mediated downregulation of *Tg*PIS perturbs PtdIns, PtdSer, and PtdThr species. Lipids isolated from the *Tg*PIS-mAID-3xHA mutant and parental strain (RHΔ*ku80*Δ*hxgprt*-TIR1) were subjected to lipidomic analysis. For each phospholipid class shown herein, the amount of all major species (accounting for >90% of total lipid) was plotted in pmol/10^6^ parasites. Numerical values depict the means with S.E. from 4 independent experiments. Samples without (–) or with (+) IAA treatments are colored differently for the parental and mutant strains. Lipid species are ordered based on their acyl chain length from the top to bottom of the graph. Changes in the contour of violin-like graphs are meant to show the overall variation in given lipids. Statistical significance was scored for each lipid species by comparing the auxin-treated and control samples (Student’s *t* test; *p ≤ 0.05; **p ≤ 0.01; ***p ≤ 0.001).

In accord with other phenotyping assays, we collected lipidomic samples after 96 h of auxin treatment. Lipid analyses of the parental and mutant strains revealed six phospholipid classes, namely PtdCho, PtdEtn, PtdSer, PtdThr, PtdIns, and PtdGro, and two sphingolipids (sphingomyelin and ethanolamine phosphorylceramide), confirming our earlier reports^3,4^ (Supplementary Figure 4c). Even though the amount of all major lipid classes remained statistically unaltered, we observed a perturbation in the levels of PtdIns, PtdSer, and PtdThr. Given a strong phenotype in the *Tg*PIS-mAID-3xHA mutant, the reduction in PtdIns seemed particularly minor, prompting us to inspect alterations in phospholipid species. We quantified the most abundant species of each lipid class in untreated and auxin-treated samples (Fig. 6, Supplementary Figure 5). As expected, the content of none of the shown lipid species was affected in the parental strain. In contrary, the mutant showed a clear shift in the specific species of PtdIns, PtdSer, and PtdThr after auxin exposure. Especially, PtdIns species with short-to-medium chains (C30, C32, C34) were highly reduced, whereas PtdIns C38 was increased, though not statistically significant. On the other hand, designated species of PtdThr (C36, C38) were significantly declined, while of PtdSer (C34, C36) were increased. Species of PtdCho, PtdEtn, and PtdGro did not change upon auxin treatment in any of the two strains (Supplementary Figure 5). The data, therefore, disclosed a selective modulation of major anionic phospholipid species following knockdown of PIS.

### Changes in anionic lipids reflect perturbed membrane stability in the PIS mutant

**Fig. 7:**
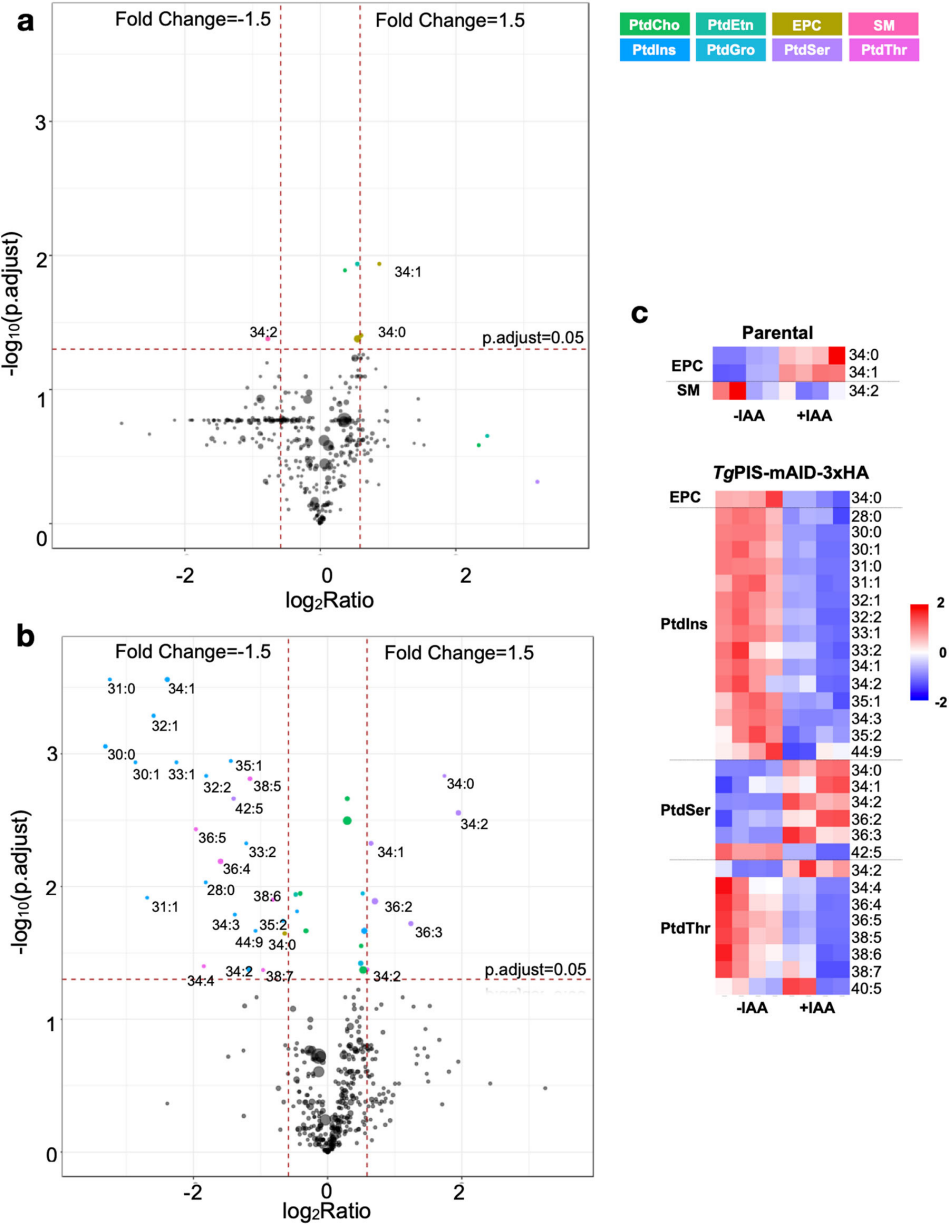
Conditional depletion of PtdIns synthase causes modulation of selected lipid species. **a**, **b** Volcano plots illustrating changes in all detectable lipid species upon IAA treatment of the parental and *Tg*PIS-mAID-3xHA strains. Thresholds of false-discovery rate (FDR)-corrected *p*-value (≤0.05) and fold-change (≥1.5) were used to define significantly-altered metabolites. Lipid species (represented as circles) are scaled to abundance; those above the threshold are colored according to phospholipid class, while others are shown in gray/black. The horizontal dashed line corresponds to statistical significance, and the two vertical dashed lines to a decrease or increase by a factor of 1.5 (*n* = 4 assays). **c** Heatmaps showing the induction or repression of lipid species chosen from **a** and **b**.

To consolidate our results, we plotted the magnitude of change in all detectable species from all shown phospholipids (irrespective of abundance) in volcano plots, which illustrate the fold-change *vs*. statistical significance in response to auxin treatment (Fig. 7a–b). The majority of lipid species in the parental strain had no perturbation except for some ethanolamine phosphorylceramide and sphingomyelin species that were affected ≥1.5-fold (*p* value ≤0.05) (Fig. 7a). The *Tg*PIS-mAID-3xHA mutant by contrast exhibited significant modulation of several species of PtdIns, PtdSer, and PtdThr above the threshold (Fig. 7b). As depicted in heatmaps (Fig. 7c), we noted evident decline in species of PtdIns and PtdThr, while the level of most PtdSer species was induced. These results encouraged us to deduce the effect of PIS mutation on the physicochemical features of the membrane lipids. In this regard, we generated violin plots based on the calculated equivalent carbon numbers (ECN) for indicated lipid classes (Supplementary Figure 6). The ECN is normally used in reversed-phase chromatography of lipids and proportional to the strength of interaction between a lipid and hydrocarbon tails of the stationary phase^54,55^. In biological membranes, hydrophobic interactions between lipids with higher ECN are stronger and vice versa, thus it can be regarded as one of the key characteristics determining the membrane stability. Indeed, the ECN density plots displayed altered contour of PtdIns, PtdThr, PtdSer, and PtdGro in auxin-treated PIS mutant but not in the parental strain, resonating with our above-mentioned analysis of individual lipid species.

### *Toxoplasma* can salvage specific PtdIns species with long acyl chains from the host cell

**Fig. 8:**
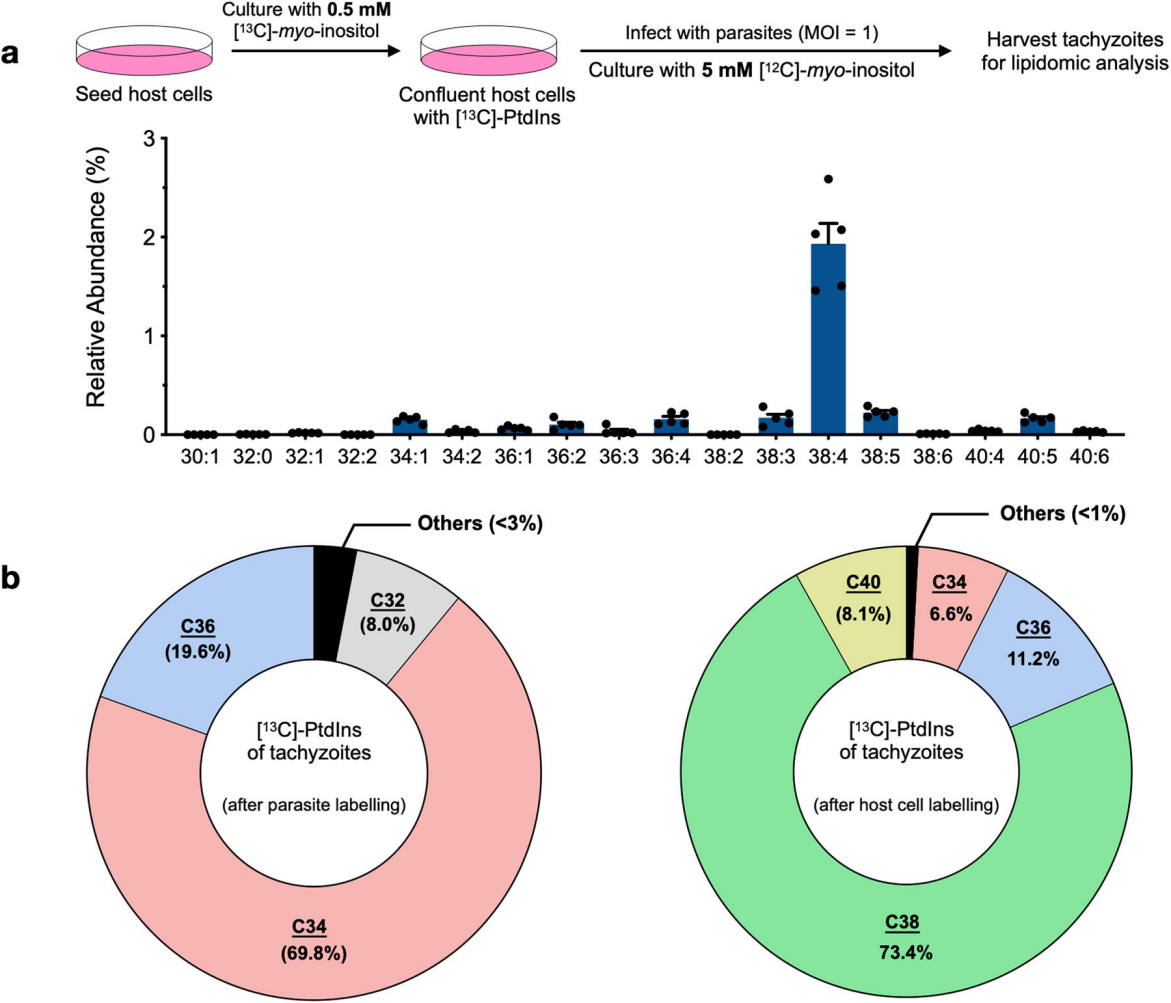
Tachyzoites can synthesize as well as salvage selected PtdIns species from host cells. **a** HFF labeling with [^13^C]-*myo*-inositol and intracellular propagation of tachyzoites in prelabeled host cells to test the salvage of [^13^C]-PtdIns. Briefly, host cells were grown to confluence in the presence of 0.5 mM [^13^C]-*myo*-inositol, and then infected with tachyzoites of the PIS mutant (MOI = 1) in the presence of 5 mM *myo*-inositol to minimize the import and usage of residual intracellular isotope by the parasite’s de novo synthesis. Purified extracellular tachyzoites were examined by lipidomic analysis. Note that this assay was technically (intricate data normalization) not feasible with the auxin-treated *Tg*PIS mutant due to a need of higher MOI for initial infection (and thus more unlabeled PtdIns), and much slower growth in the presence of IAA (and thus prolonged incubation). The data plotted are from 5 independent assays (mean ± S.E.). **b** Proportion of [^13^C]-PtdIns species detected after stable isotope labeling of extracellular tachyzoites (left, see Fig. 4e), and in tachyzoites after propagation in prelabelled host cells (right, see **a**). Only primarily-labeled PtdIns species (≥1%) are named; the minor-labeled species are shown as “Others”.

One intriguing observation in the lipidomic profiling of the PIS mutant was a somewhat selective increase in an abundant species of PtdIns with long acyl chain (C38), which counterbalanced the decline in most other species with shorter chains, and perhaps also offset the expected defects in motility, invasion and egress (see “Discussion”). Further, despite the marked presence of long-chain PtdIns species in parasites (Figs. 6 and 7), the isotope labeling of extracellular tachyzoites resulted in only insignificant inclusion of tracer in such lipid species (Fig. 4e). These unexpected findings led us to investigate the potential salvage of PtdIns from host cells. We first labeled the human fibroblasts with [^13^C]-*myo*-inositol and then infected them for propagating parasites (Fig. 8a). Lipidomics of the tachyzoite progeny isolated from prelabeled host cells revealed rather selective accrual of C38:4 PtdIns. A comparison of [^13^C]-PtdIns in tachyzoites that were labeled extracellularly (host-free) versus those grown in pre-labeled host cells clearly indicated distinct profiles of tracer incorporation (Fig. 8b). While the former displayed a majority of labeling in C34 PtdIns (70%), followed by C36 (20%) and C32 (8%), the latter parasites had primarily C38 (73%), C36 (11%), C40 (8%) and C34 (7%) species. Collectively, our lipidomic and isotope labeling results strongly suggest that *T. gondii* can produce PtdIns species with short-medium acyl chains de novo, while at the same time the parasite can salvage longer-chain lipid species from the host cell.

## Discussion

**Fig. 9:**
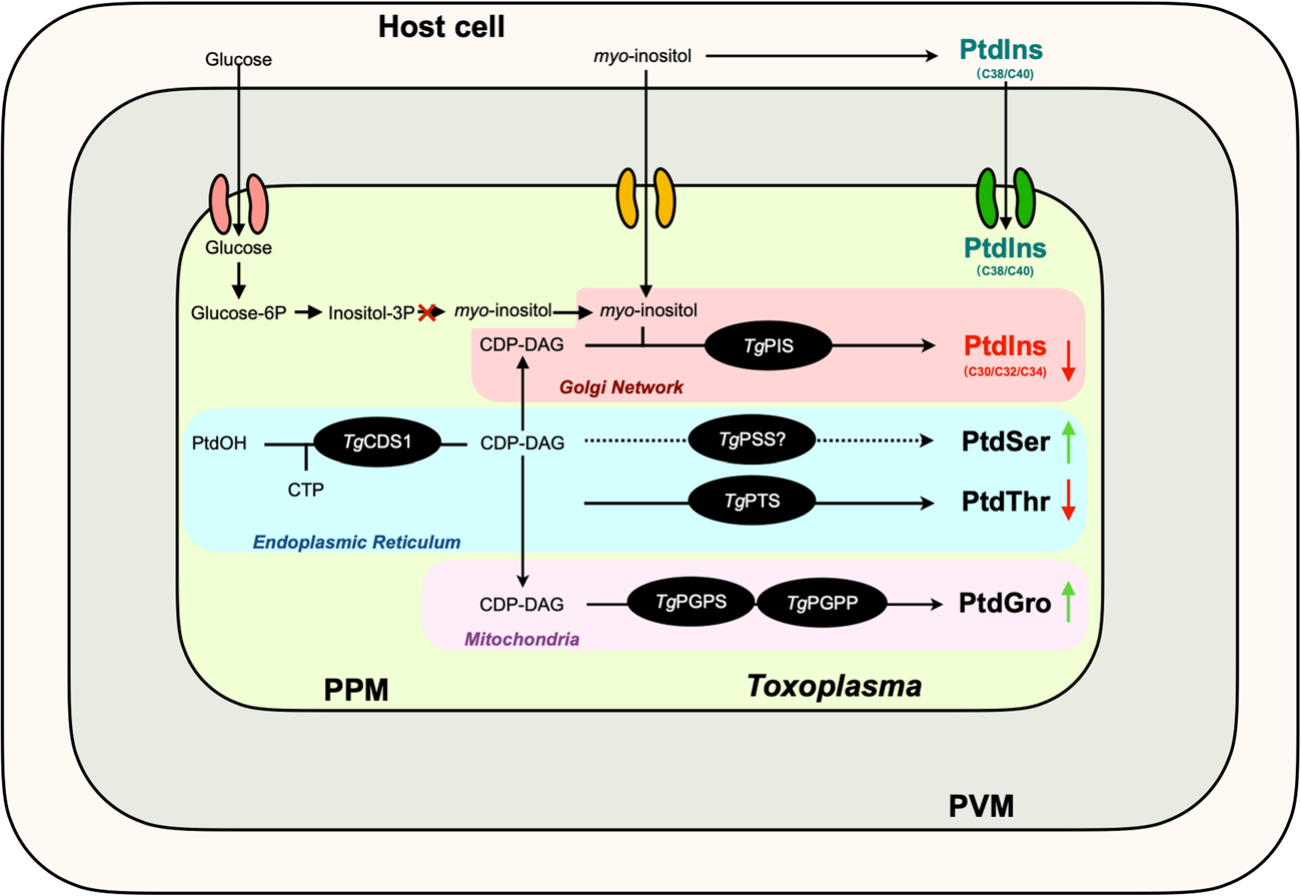
Model of PtdIns biosynthesis and homeostasis of anionic phospholipids in *T. gondii*. PtdIns is made by *Tg*PIS in the Golgi network using CDP-DAG and *myo*-inositol. CDP-DAG is generated by *Tg*CDS1 in the endoplasmic reticulum, whereas *myo*-inositol is imported from the milieu. De novo synthesis of *myo*-inositol involves conversion of glucose to glucose-6-phosphate by hexokinase, followed by the action of inositol-3P synthase to produce inositol-3-phosphate. Successively, Ins3P is dephosphorylated to make inositol by inositol-3P monophosphatase. There is no biochemical and genetic evidence for the latter enzyme in *T. gondii*. Knockdown of *Tg*PIS reduces the level of many PtdIns species along with homeostatic modulation of other anionic lipids (namely PtdThr, PtdSer, PtdGro species). PtdIns species with shorter chains (e.g., C30/C32/C34) are synthesized de novo, while PtdIns species with relatively longer acyl chain (C38/C40) are salvaged by the parasite from its host cell. Based on isotope labeling studies, we speculate that both routes contribute to the synthesis of C36 PtdIns species (not shown). Transporters, inter-organelle contact sites, or other lipid trafficking pathways, as stated, enable transport of *myo*-inositol and CDP-DAG. The question-marked reaction specifies yet-unknown enzyme involved in CDP-DAG-dependent PtdSer synthesis. Selected abbreviations: CDS, CDP-DAG synthase; PGPS, PtdGro phosphate synthase; PGPP, PtdGro phosphate phosphatase; PSS, PtdSer synthase; PTS, PtdThr synthase; PPM, parasite’s plasma membrane; PVM, parasitophorous vacuole membrane.

The enzyme PIS, utilizing CDP-DAG and *myo*-inositol to produce PtdIns, is conserved in all eukaryotes including in *T. gondii* (Fig. 9). Many protozoan parasites harbor all three enzymes to produce *myo*-inositol from glucose besides the sugar transporters, which have been characterized in *Trypanosoma* and *Leishmania*^56–58^. In *T. brucei*, endogenously-synthesized *myo*-inositol supports the synthesis of PtdIns and GPI in the ER^40,41^, while *myo*-inositol acquired from the milieu drives the bulk production of PtdIns in the Golgi complex^42,43^. Similarly, *P. falciparum* is dependent on de novo *myo*-inositol synthesis for GPI assembly^59^. Intriguingly, one of the genes encoding for inositol-3-phosphate synthase could not be identified in the genome database of *T. gondii*, and tachyzoites cultivated with [^13^C]-glucose showed the labeling of glucose-6-phosphate but not of inositol-3-phosphate and *myo*-inositol^60^. Equally, we reveal that tachyzoites import and utilize *myo*-inositol for PtdIns synthesis. Our earlier work identified four sugar transporters in *T. gondii*, of which two reside in the plasma membrane^61^. One of them, *Tg*GT1, can transport glucose, mannose, fructose, and galactose, while the other (*Tg*ST2) may facilitate *myo*-inositol import. Surprisingly though, *Tg*ST2 is dispensable for the lytic cycle of tachyzoites; hence it remains equivocal whether the parasite is indeed strictly dependent on (auxotrophic for) host-derived *myo*-inositol (Fig. 9).

The other precursor of PtdIns, CDP-DAG, is synthesized by the enzyme CDS using phosphatidic acid and CTP (Fig. 9). The eukaryotic-type CDSs have been identified in the genomes of many protozoan parasites, and proved to be essential for the synthesis of PtdIns in *T. gondii*, *P. falciparum,* and *T. brucei*^7,62–64^. Additionally, we found a prokaryotic-type CDS in *T. gondii* (and other selected parasites) that drives the synthesis of phosphatidylglycerol^7^. This study shows that a knockdown of CDS1 in the ER of *T. gondii* impaired the incorporation of [^3^H]-*myo*-inositol into PtdIns, and caused an equivalent decrease in the lipid content of the CDS1 mutant. Albeit a dependence of PtdIns synthesis on other CDP-DAG pools cannot be entirely ruled out, it can be concluded that the ER-derived CDP-DAG serves as a key source for PtdIns synthesis in the Golgi network, which would necessitate its transport from the ER to Golgi (Fig. 9). Subsequently, PtdIns should be transported from Golgi to the site of GPI assembly^65^. The mechanism of such inter-organelle exchange of CDP-DAG and PtdIns warrant further investigation.

Autonomous synthesis, trafficking, and physiological relevance of membrane phospholipids are active areas of research in protozoan parasites^2^. In *T. gondii*, de novo synthesis of PtdCho is critical for normal progression of the lytic cycle, because the parasite seems unable to import sufficient amount of lipid or certain species from the host cells to bypass the ablation of its CDP-choline pathway^5,8^. Likewise, the endogenous synthesis of PtdThr—a rare coccidian-specific lipid—is critical for the growth and virulence of tachyzoites. In contrast, the parasite shows significant plasticity in synthesis of PtdEtn, which can occur in multiple organelles^6,9^. This study revealed a vital role of PtdIns synthesis despite the fact that tachyzoites are competent in importing some host-derived lipid species. Using post-translational control of protein stability by auxin, not only were we able to conclude a critical function of PtdIns synthesis for the cell division, gliding motility, invasion, and egress, but also perform comprehensive lipidomic and isotope-labeling analyses, unveiling additional noteworthy findings. These include de novo synthesis of PtdIns, salvage of specific host-derived lipid species, and integrated regulation of major anionic glycerophospholipids in *T. gondii* (Fig. 9).

A reduction in PtdIns species with short-medium acyl chains (C30, C32, C34) and an increase in lipid species with long-chain (C38) following a knockdown of PIS correlate well with our isotope labeling assays. A few abundant species of PtdIns were either not affected or even slightly increased upon knockdown of *Tg*PIS, balancing the content of PtdIns. Nonetheless, ablation of de novo synthesis led to a severe phenotype, which can be attributed to a requirement of short-to-medium chain PtdIns species for the membrane biogenesis during endodyogeny. Besides endogenously made lipid species, the parasite salvages (at least) C38:4 PtdIns from the host cell. Markedly, this particular lipid is found to be enriched in human host cells used herein to propagate tachyzoites^66^. Moreover, C38:4 is the dominant PtdIns species that drives the synthesis of phosphoinositides in mammalian cells^67,68^. Thus, we speculate that scavenging of such lipid species from the host cell may enable the parasite to generate adequate phosphoinositides for operating its own IP_3_-dependent Ca^2+^ signaling, which is known to govern the motility, egress, and invasion processes^16^. Taken together, our phenotypic and lipidomic findings advocate the usage of distinctive PtdIns species for membrane biogenesis and signaling in tachyzoites of *T. gondii*.

Last but not least, we observed a rather specific homeostasis of PtdIns, PtdThr, PtdSer, and PtdGro in the PIS mutant, suggesting inter-regulation of their biosynthesis (Fig. 9). Increased levels of PtdSer and PtdGro can be explained by rerouting of excess CDP-DAG after reduced PtdIns synthesis. A decrease in PtdThr on the other hand may be a consequence of its involvement in calcium homeostasis^10,11^, and/or due to its inversely-proportional relationship with the PtdSer content, as also observed in other mutants, where PtdThr and PtdSer syntheses were impaired^4^. A balanced composition of anionic phospholipids, accounting for up to 30% of membrane lipids in eukaryotic cells^69^, is needed for optimal function of many enzymes, e.g., protein kinase C, phospholipase A2, and CTP-phosphocholine cytidylyltransferase^70^. It has been proposed that the negatively charged region of the membrane interacts with the cationic motif of proteins^71,72^, and the head groups and acyl chains are determinant factors for such interactions^70^. Anionic lipids are also critical for the functioning of membrane permeases, such as glucose transporters and ATP-sensitive potassium channels in mammalian cells^73,74^. Given these and our principal findings on anionic phospholipids, future work should examine their importance and mechanistic roles in *T. gondii*.

## Methods

### Biological reagents and resources

The RHΔ*ku80*Δ*hxgprt*^75^ and RHΔ*ku80*Δ*hxgprt*-TIR1^49^ strains of *T. gondii* were kindly offered by Vern Carruthers (University of Michigan, MI) and David Sibley (Washington State University, St. Louis, MO), respectively. The Δ*tgcds1*_*r*_ mutant was generated in our earlier study^7^. The *pG140* plasmid was donated by Markus Meissner (Ludwig-Maximilians University, Munich). Antibodies recognizing the *Tg*HSP90, *Tg*GAP45, *Tg*PDH-E1a, *Tg*SAG2, and *Tg*IMC3 proteins were bestowed by Sergio Angel (IIB-INTECH, Argentina), Dominique Soldati-Favre (University of Geneva, Switzerland), Bang Shen (Huazhong Agricultural University, China), Honglin Jia (Harbin Veterinary Research Institute, China) and Marc-Jan Gubbels (Boston College, MA), respectively. Anti-*Tg*SERCA and anti-*Tg*PSD1_mt_ antisera were obtained from FriendBio Bioscience and Technology (China). Other primary antibodies recognizing the *Tg*SAG1 protein and engineered epitopes (HA and Ty1) were obtained from ThermoFisher Scientific and Sigma-Aldrich (Germany). Corresponding secondary antibodies (Alexa488, Alexa594) were procured from Life Technologies (Germany). [^3^H]-*myo*-inositol, and silica plates for TLC were obtained from American Radiolabeled Chemicals (St. Louis, MO) and Merck Millipore (Billerica, MA), respectively. [^13^C]-*myo*-inositol was kindly provided by Dorothea Fiedler (Leibniz Research Institute, Berlin). Lipid standards and 1,2-dioleoyl-sn-glycero-3-(cytidine diphosphate) (CDP-DAG) were delivered by Avanti Polar Lipids (USA). DNA oligonucleotides (Supplementary Table 2) were purchased from Life Technologies.

### Parasite and host cell culture

Tachyzoites of the aforementioned *T. gondii* strains were propagated by serial passaging in confluent human foreskin fibroblasts (HFFs; Cell Lines Service, Eppelheim, Germany) using a routine multiplicity of infection (MOI) of 3. Uninfected host cells were grown to confluence in flasks, dishes, or plates as required, and harvested for further passaging by trypsin-EDTA treatment. Parasitized as well as infected HFFs were cultured in Dulbecco’s Modified Eagle’s Medium (DMEM) supplemented with glucose (4.5 g/L), fetal bovine serum (10%, PAN Biotech, Germany), glutamine (2 mM), sodium pyruvate (1 mM), penicillin (100 U/mL), streptomycin (100 μg/mL) and minimum Eagle’s non-essential amino acids (100 μM of each, serine, glycine, alanine, asparagine, aspartic acid, glutamate, and proline) in a humidified incubator (5% CO_2_, 37 °C). For most assays, parasites were mechanically released from the late-stage cultures. Briefly, parasitized cells were scraped and squirted through 23- and 27-gauge syringes to obtain extracellular tachyzoites, which were utilized directly for transfection and phenotyping assays. For biochemical analyses, fresh parasites were washed twice with phosphate buffer saline (PBS) by centrifugation (400 *g*, 10 min, 4 °C), filtered (5 μm) to remove the host-cell debris, and then subjected directly to metabolic labeling, or alternatively stored at −80 °C for lipid, protein, and nucleic acid analyses.

### Radioisotope labeling of extracellular parasites

The metabolic labeling of tachyzoites was performed as described elsewhere^5^. Briefly, fresh extracellular tachyzoites of the RH or Δ*tgcds1*_*r*_ strains (0.5 − 1 × 10^8^ cells) were incubated with [^3^H]-*myo*-inositol (10 µCi, 0.025 or 0.5 mM) in the absence or presence of 1,2-dioleoyl-CDP-DAG (dried freshly in the reaction tube and vortexed) in 1 mL medium (pH 7.4). The labeling medium contained HEPES (20 mM), KCl (140 mM), NaCl (10 mM), MgCl_2_ (2.5 mM), CaCl_2_ (0.1 μM), sodium pyruvate (1 mM), glucose (5 mM), Mg-ATP (1 mM), MEM vitamins (1x), MEM amino acids and non-essential amino acids. The non-essential amino acid solution included alanine, aspartate, glutamate, glycine, proline, and asparagine (10 μg/mL each). ATP and glucose solutions were prepared fresh and added to a final concentration, as indicated, prior to starting the reaction. The assay was executed in glass tubes at 37 °C on a shaking water bath for the time periods specified in the respective figures. The reaction was terminated by adding chloroform and methanol (1.1 mL each), followed by vortexing and centrifugation (1000 *g*, 10 min). The lower chloroform phase of the resultant biphasic system was used to recover phospholipids for TLC or quantification of the radioactive tracer by liquid scintillation counting.

### Stable isotope labeling of extracellular and intracellular tachyzoites

For [^13^C]-*myo*-inositol labeling of extracellular parasites, the *Tg*PIS-mAID-3xHA mutant was precultured without or with 500 μM IAA for 90 h, followed by mechanical (syringe)-release, PBS washing, and filtering through 5 μm filter to remove the host cell debris. Tachyzoites (1 × 10^7^) were suspended in 100 μL DMEM containing 0.5 mM [^13^C]-*myo*-inositol in the absence or presence of IAA, and incubated at 37 °C for 6 h prior to lipidomic analysis. For the intracellular labeling of tachyzoites in HFF cells prelabeled with [^13^C]-PtdIns, host cells were seeded in T-175 flask and grown to confluence in the presence of 0.5 mM [^13^C]-*myo*-inositol. They were washed twice with PBS to remove the unincorporated [^13^C]-*myo*-inositol, and then infected with parasites in the presence of 10-fold excess (5 mM) of normal *myo*-inositol (Sigma-Aldrich, Germany) to minimalize the incorporation of residual intracellularly-accumulated isotope into de novo-synthesized PtdIns of the proliferating parasites. The progeny parasites were subjected to lipidomic analysis.

### Molecular cloning

RNA was isolated from fresh extracellular parasites using TRIzol-extraction method and subsequently reverse-transcribed into first-strand cDNA (Life Technologies, Germany). Genomic DNA was prepared using a commercial kit (Jena Bioscience, Germany). Mutagenesis of the conserved residues in *Tg*PIS sequence was performed using the Q5 site-directed mutagenesis kit (New England Biolabs, MA). The open reading frames of *Tg*PIS^Δ91–100^ and *Tg*PIS^Δ108–117^ were synthesized by GenScript (USA). All amplicons were PCR-amplified using the Pfu Ultra II fusion HS DNA polymerase (Agilent Technologies, CA), and cloned into pertinent vectors by either restriction enzyme-mediated or ligation-independent cloning, as indicated elsewhere in this work. Plasmids were transformed into XL-1b strain of *Escherichia coli* for molecular cloning and vector amplification.

### Functional expression in *Escherichia coli*

The open reading frames of *Tg*PIS and mutants were cloned into the *pQE60* expression vector (Qiagen, Germany) at *Bgl*II restriction site and then transformed into M15/pREP4 strain of *E. coli*. The bacterial strains expressing the wild-type *Tg*PIS or its specified mutants, as well as the negative control strain harboring empty vector were cultured in M9 minimal medium containing ampicillin (100 mg/L) and kanamycin (50 mg/L). Protein expression was induced overnight at 25 °C by adding 1 mM Isopropyl β-D-1-thiogalactopyranoside (IPTG) to the cultures with OD_600_ of 0.4–0.6 (pre-grown at 37 °C), followed by additional incubation with *myo*-inositol at 37 °C for 4 h. Samples were processed for lipids extraction and TLC.

### Lipid extraction, TLC separation, and quantification

Lipids were extracted according to our previously reported protocol^5^ based on the original method of Bligh and Dyer^76^. Briefly, cell pellets were suspended in 4 mL methanol:water (2:0.9, v/v), followed by sequential addition of chloroform (2 mL), 0.2 M KCl (1.8 mL), and chloroform (2 mL), each accompanied with vigorous vortex-mixing. The aqueous upper phase was removed from the biphasic system, and the lower chloroform phase was backwashed twice with 2.1 mL of methanol:KCl (0.2 M):chloroform (1:0.9:0.1, v/v). Lipids obtained from the isotope-labeled parasites or from recombinant bacteria were backwashed 3x, each with 2.1 mL of methanol:PBS:chloroform (1:0.9:0.15, v/v). The chloroform phase containing lipids was recovered, dried under N_2_ stream, and resuspended in 50–100 μL of chloroform:methanol (9:1).

Phospholipids were resolved by one-dimensional TLC on silica H plates developed either in a solvent comprising chloroform, methanol, 2-propanol, KCl (0.25%), and triethylamine (90:28:75:18:54, v/v), or chloroform, ethanol, water, and triethylamine (30:35:7:35, v/v). Alternatively, lipids were separated by two-dimensional TLC on silica 60 plates developed first in chloroform, methanol, and NH_4_OH (65:35:5, v/v), and then in chloroform, acetic acid, methanol, and water (75:25:5:2.2, v/v). They were visualized by incubating the TLC plate in a glass chamber with iodine vapor, and/or by spraying 0.2% (w/v) anilino-1-naphthalene sulfonic acid followed by ultraviolet light exposure, or by autoradiography using Fuji X-ray films. Phospholipids were identified based on their comigration with authentic standards. TLC-resolved silica scrapings were assayed to quantify individual phospholipids by lipid-phosphorus measurements using defined chemical standards, as described elsewhere^77^.

### Lipidomic analysis

Pellets of purified parasites (1–2 × 10^7^) were suspended in 0.8 mL PBS and subjected to lipid extraction according to Bligh and Dyer^76^. Lipid extracts were dried under N_2_, dissolved in 100 μL of chloroform and methanol (1:1), and injected (10 μL) into a hydrophilic interaction liquid chromatography column (2.6 μm HILIC 100 Å, 50 × 4.6 mm, Phenomenex, CA). Lipid classes were separated by gradient elution on an Infinity II 1290 UPLC (Agilent, CA) at a flow rate of 1 mL/min. Acetonitrile and acetone (9:1, v/v) with 0.1% formic acid was used as solvent A, while solvent B consisted of a mixture of acetonitrile, H_2_O (7:3, v/v), 10 mM ammonium formate, and 0.1% formic acid. Gradient elution was done as follows (time in min, % B): (0, 0), (1, 50), (3, 50), (3.1, 100), (4, 100). No re-equilibration of the column was necessary between successive samples. The column effluent was connected to a heated electrospray ionization (hESI) source of either an Orbitrap Fusion or Q Exactive HF mass spectrometer (Thermo Scientific, MA) operated at −3600 V in the negative ionization mode. The vaporizer and ion transfer tube were set at a temperature of 275 °C and 380 °C, respectively.

Full scan measurements (MS1) in the mass range from 450 to 1150 amu were collected at a resolution of 120000. Parallelized data-dependent MS2 scans were done with HCD fragmentation set at 30 V, using the dual-stage linear ion trap to generate up to 30 spectra/second. The data processing using R script was based on the packages, *XCMS* for peak recognition and integration, and *pcaMethods* and *statistics* for statistical analysis. Lipids were identified by matching the retention time and mass of experimental data to the lipid database, containing both labeled and unlabeled lipids. Quantification was achieved by calculating response factors for individual lipid classes using standards, when available.

### Making of transgenic parasites

The plasmid constructs (20–50 μg) were transfected into freshly isolated tachyzoites of specific strains (~10^7^ cells) suspended in filter-sterile Cytomix (120 mM KCl, 0.15 mM CaCl_2_, 10 mM K_2_HPO_4_/KH_2_PO_4_, 25 mM HEPES, 2 mM EGTA, 5 mM MgCl_2_ supplemented with fresh 5 mM glutathione and 5 mM ATP; pH 7.6) using a BTX electroporation instrument (2 kV, 50 Ω, 25 μF, 250 μs). Transgenic parasites were selected for resistance to a drug corresponding to the selection marker encoded by the transfected plasmid. The stable drug-resistant parasites were cloned by limiting dilution, and individual clones were screened by PCR and/or immunostaining assays. The constructs, primers, and the parasite strains along with other relevant details are described in Supplementary Table 2 and respective figure legends. To express *Tg*PIS^49–258^-HA (Fig. 2f), its open reading frame was cloned into the *pTETO7SAG1-UPKO* plasmid at *Nco*I and *Pac*I sites. The construct was linearized by *Not*I enzyme and transfected into the RHΔ*ku80*-TaTi strain, followed by negative selection for the disruption of the *UPRT* locus using 5-fluorodeoxyuridine (5 μM)^78^. The resultant strain expressing *Tg*PIS^49–258^-HA was afterward transfected with a vector encoding *Tg*ERD2-Ty1 (Golgi marker) for co-localization assays.

To create the Δ*tgpis*-HA_Excised_ mutant (Fig. 3), the HA-tagged open reading frame, 5′UTR and 3′UTR of *Tg*PIS were cloned into the *pG140* vector at the *Apa*I/*EcoR*I, *EcoR*I/*Pac*I, and *Sac*I sites, respectively. The construct was linearized by *Apa*I and transfected into the RHΔ*ku80*Δ*hxgprt* strain. Transgenic parasites were selected for the HXGPRT expression using mycophenolic acid (25 μg/mL) and xanthine (50 μg/mL)^79^. The eventual *Tg*PIS-HA_Floxed_ strain expressed loxP-flanked *Tg*PIS-HA regulated by native promoter and *Tg*DHFR-TS-3′UTR. Parasites were consequently transformed with the *pSAG1-Cre* plasmid to induce Cre-mediated recombination for deleting *Tg*PIS-HA while concurrently repositioning YFP directly after the *Tg*PIS promoter, which in turn yielded a yellow-fluorescent Δ*tgpis*-HA_Excised_ mutant. For making the *Tg*PIS-mAID-3xHA mutant (Fig. 4), we designed *pU6-Cas9-TgPIS*_*sgRNA*_ construct expressing Cas9 (under *Tg*TUB1 promoter), and *sg*RNA targeting the 3′UTR of *TgPIS*. It was co-transfected with a donor amplicon, comprising mAID-3xHA-*Tg*GRA1–3′UTR and HXGPRT marker flanked by short (40 bp) 5′ and 3′ homology arms for crossover at the *TgPIS* locus, in the RHΔ*ku80*Δ*hxgprt*-TIR1 strain. Parasites were selected using mycophenolic acid (25 μg/mL) and xanthine (50 μg/mL)^79^. The method allowed 3′-genomic tagging of the PIS protein with mAID-3xHA and its conditional expression (auxin-regulated) under the control of the native promoter and *Tg*GRA1–3′UTR.

### Indirect immunofluorescence assays

The assay was performed essentially as described earlier^80,81^. Parasitized HFFs grown on coverslips (24–40 h infection) were washed with PBS, fixed with 4% paraformaldehyde (10 min), and neutralized with 0.1 M glycine in PBS (5 min). Cells were permeabilized by 0.2% Triton X-100 in PBS (20 min), and then treated with 2% bovine serum albumin (BSA) dissolved in 0.2% Triton X-100/PBS for 20 min. Samples were stained with primary antibodies (α-HA, mouse or rabbit, 1:1000 or 1:3000; α-Ty1, mouse hybridoma, 1:50; α-*Tg*GAP45, rabbit, 1:10000; α-*Tg*PDH-E1a, rabbit, 1:1000; α-*Tg*IMC3, rabbit, 1:2000; α-*Tg*SERCA, rabbit, 1:100; α-*Tg*PSD1_mt_, rabbit, 1:100; α-*Tg*SAG1, mouse, 1:10000; α-*Tg*SAG2, rabbit, 1:1000) for 1 h. Cells were washed three times with 0.2% Triton X-100 in PBS, and stained with Alexa488/594-conjugated secondary antibodies for 45 min. Following additional washing with PBS, samples were mounted in Fluoromount G and DAPI mixture (Southern Biotech, Birmingham, AL) and stored at 4 °C. Images were acquired by fluorescence microscopy (Zeiss, Germany).

### Immunoblot assays

Tachyzoites (1–2 × 10^7^) were washed twice with PBS, pelleted (400 *g*, 10 min, 4 °C), resuspended in the Laemmli protein-loading buffer and subjected to denaturing gel electrophoresis. Proteins were resolved by 12% SDS-PAGE and transferred onto a nitrocellulose membrane (85 mA, 90 min). The blot was treated overnight (4 °C) with 5% skimmed milk suspended in Tris-buffered saline (20 mM Tris base and 150 mM NaCl, 0.2% Tween 20, pH 7.4), incubated with α-HA (1:1000, mouse) and α-*Tg*HSP90 (1:1000, rabbit) antibodies for 2 h, washed 3x (5 min each), and then incubated with infrared dyes-conjugated secondary antibodies (680RD and 800CW, each at 1:10000 dilution for 1 h). Proteins were visualized using a Li-COR imaging system (Li-COR Biosciences, USA).

### Lytic cycle assays

Standard phenotyping methods were used to determine the impact of genetic manipulation on the lytic cycle of tachyzoites in vitro, as described earlier^10,53^. Plaque assays were performed by infecting confluent HFF cells in 6-well plates (100–200 parasites/well). Infected cells were incubated unperturbed for 7 days, followed by fixation with ice-cold methanol and staining with crystal violet (5%). Plaques were imaged and scored for size and number using ImageJ software (NIH, Bethesda, MD). For invasion assays, confluent HFF cells cultured on coverslips, were infected with tachyzoites (MOI: 10) for 1 h. Cultures were stained with α-*Tg*SAG1 antibody (mouse, 1:10000) prior to detergent permeabilization to visualize the noninvaded or extracellular parasites. Cells were washed 3x with PBS, permeabilized with 0.2% Triton X-100 in PBS for 20 min, and then stained with α-*Tg*GAP45 antibody (rabbit, 1:10000) to identify invaded parasites. The fractions of invaded (intracellular) parasites were determined across the strains to compare their invasion efficiency.

To measure the gliding motility, parasites (4 × 10^5^) suspended in Hank’s balanced salt solution (HBSS) were incubated to let them settle (15 min, room temperature) and glide (15 min, 37 °C) onto 0.01% BSA-coated coverslips. Samples were stained with α-*Tg*SAG1 and Alexa488 antibodies, as mentioned above. The motile fraction was counted on the microscope and trail lengths were quantified by ImageJ software. To test the natural egress, host cell monolayers on coverslips were infected with MOI of 1 for 40 and 64 h. Similarly, induced egress in response to zaprinast treatment (500 µM) was monitored for 30 min in early cultures (MOI: 1, 24 h post-infection). In both cases, cells were fixed with 4% paraformaldehyde (15 min), neutralized by 0.1 M glycine/PBS (5 min), and blocked with 3% BSA/PBS (30 min). Egressed vacuoles were immunostained with α-*Tg*SAG1 antibody (mouse, 1:10000, 1 h) prior to permeabilization. Cultures were then washed 3 times with PBS and permeabilized by 0.2% Triton X 100/PBS (20 min), followed by staining with α-*Tg*GAP45 antibody (rabbit, 1:10000, 1 h) to visualize the intact vacuoles. Samples were washed and stained with Alexa488 and Alexa594-conjugated antibodies (1:3000, 1 h). Egress was calculated as the ratio of lysed and total vacuoles.

To gauge the cell division, confluent host cells were infected with tachyzoites (MOI: 1) and stained with α-*Tg*GAP45 antibody. The fraction of parasitophorous vacuoles containing variable numbers of parasites was determined to examine the phenotype. To compare the intracellular proliferation rates of the *Tg*PIS-HA_Floxed_ (expressing *Tg*PIS-HA) and Δ*tgpis*-HA_Excised_ (expressing YFP) parasites, the former strain was transfected with the *pSAG1-Cre* plasmid, and then used to infect confluent HFFs (MOI: 1). Samples were fixed at indicated time points and stained by α-*Tg*GAP45 antibody. The percentage of the parasitophorous vacuoles with YFP or HA signals, as well as the number of parasites/vacuoles were quantified for each time point. The budding of parasites (endodyogeny) was scored by staining with α-*Tg*IMC3 antibody (rabbit, 1:2000), as schematized in the figure and described in the figure legend.

### Statistics and reproducibility

All data shown in graphs are presented as the mean with S.E. from at least three independent assays, unless specified otherwise. Statistical analyses were performed using GraphPad Prism software (v5). Significance was tested by unpaired two-tailed Student’s *t* test with equal variance (*p ≤ 0.05, **p ≤ 0.01, ***p ≤ 0.001). In multiple comparison testing, we introduced false-discovery-rate (FDR) for correction.

### Reporting Summary

Further information on research design is available in the Nature Research Reporting Summary linked to this article.

## Supplementary information

Supplementary Information

Description of Additional Supplementary Files

Supplementary Data 1

Supplementary Data 2

Supplementary Data 3

Reporting Summary

## Data Availability

The data generated or analyzed during this study including uncropped gel/blot images are provided in the main article and associated figure files. The source data for the graphs and charts presented herein can be found in Supplementary Data 1. The lipidomic results are also furnished as Supplementary Data 2 and Supplementary Data 3. All resources are available from the authors upon reasonable request.
