# Supplementary material for: Phosphatidylinositol synthesis, its selective salvage, and inter-regulation of anionic phospholipids in Toxoplasma gondii
Source: Commun Biol. 2020 Dec 10;3:750. doi: 10.1038/s42003-020-01480-5 (PMC7728818; doi:10.1038/s42003-020-01480-5)

# Supplementary Figure 1

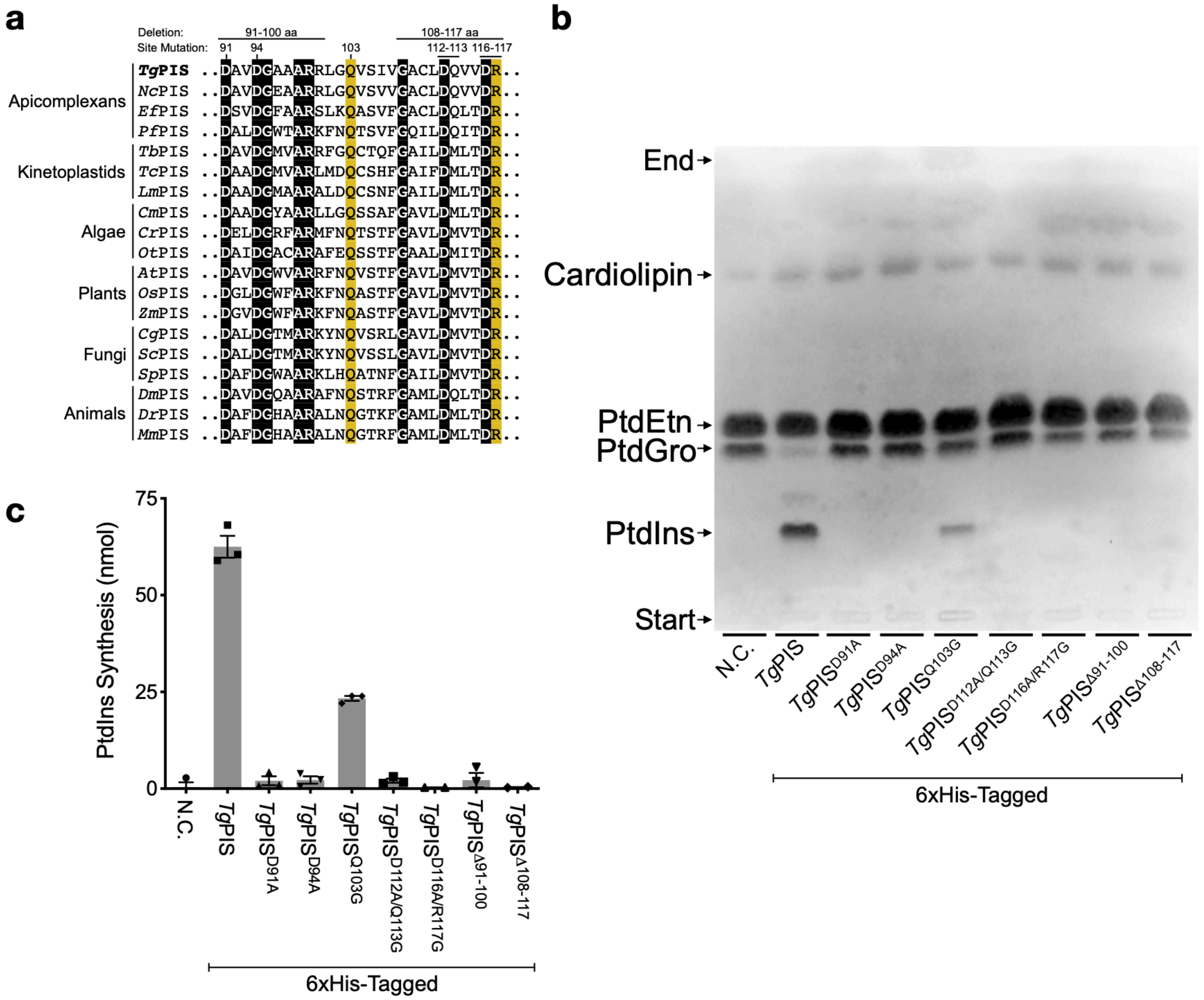


**CDP-alcohol phosphotransferase motif is essential for the catalytic activity of *Tg*PIS. (a)** Alignment of the CDP-alcohol phosphotransferase motifs present in PIS sequences. The residues conserved only in PIS sequences are highlighted as *yellow*, whereas those that are identical across all CDP-alcohol phosphotransferase domain-containing proteins are shaded in *black* (also see Supplementary Table 1). The amino acids chosen for site-directed mutagenesis or deletion are marked for their position on top. Organism abbreviations: *Nc*, *Neospora caninum*; *Ef*, *Eimeria falciformis*; *Pb*, *Plasmodium berghei*; *Pf*, *Plasmodium falciparum*; *Tb*, *Trypanosoma brucei*; *Tc*, *Trypanosoma cruzi*; *Lm*, *Leishmania major*; *Cm*, *Cyanidioschyzon merolae*; *Cr*, *Chlamydomonas reinhardtii*; *Ot*, *Ostreococcus tauri*; *At*, *Arabidopsis thaliana*; *Os*, *Oryza sativa*; *Zm*, *Zea mays*; *Cg*, *Candida glabrata*; *Sc*, *Saccharomyces cerevisiae*; *Sp*, *Schizosaccharomyces pombe*; *Dm*, *Drosophila melanogaster*; *Dr*, *Danio rerio*; *Hs*, *Homo sapiens*. **(b)** TLC-resolved lipid profile of *E. coli* M15/ pREP4 strains harboring *pQE60* (N.C., negative control), or *pQE60* constructs expressing the 6xHis-tagged *TgPIS* and its mutated variants, as indicated. Bacterial cultures were cultivated with 1 mM *myo*-inositol as described in *methods*. **(c)** Phosphorus analysis of TLC-scraped PtdIns-containing silica bands from *panel b* (n = 3 assays, mean ± S.E.).

## Supplementary Figure 2

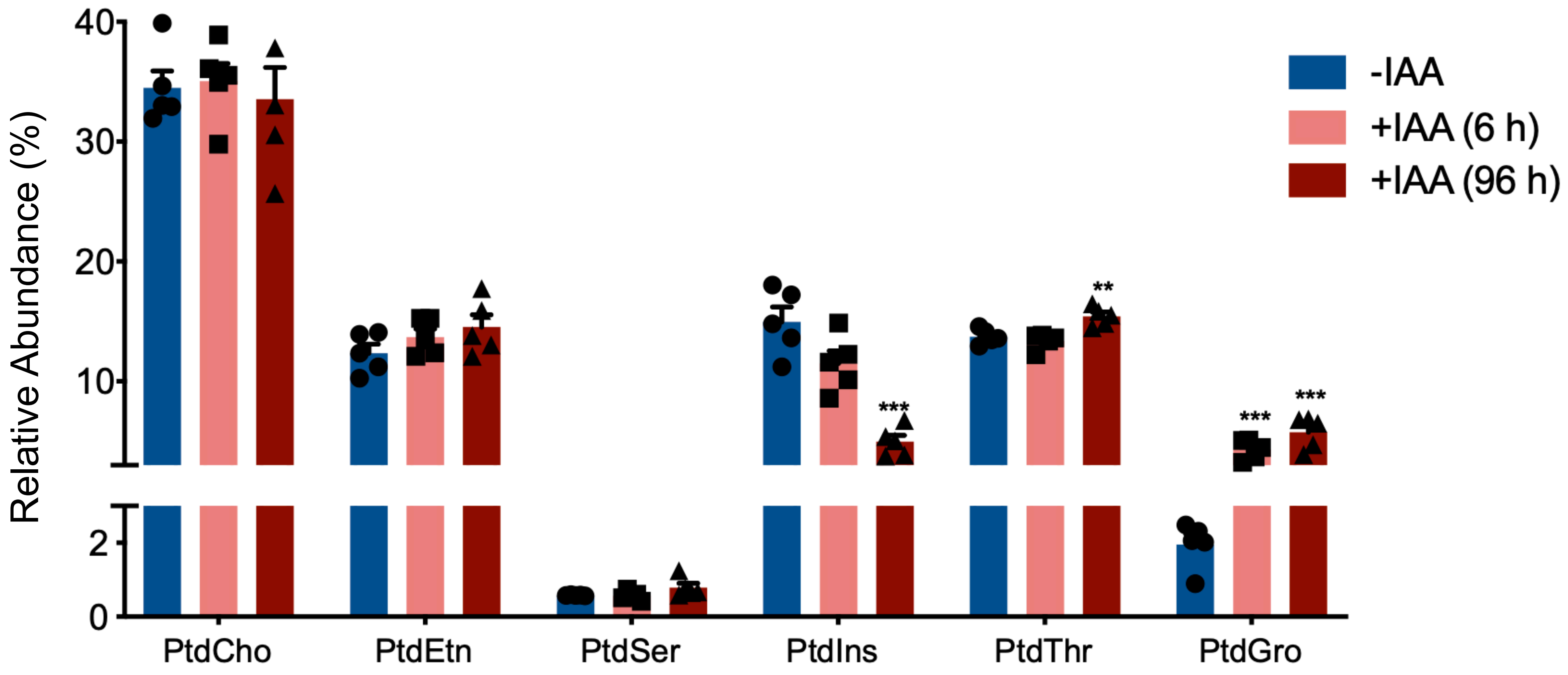


**Phospholipid profile of the PIS mutant labeled with [$^{13}$C]-*myo*-inositol.** Major phospholipids detected in the extracellular tachyzoites of the *Tg*PIS-mAID-3xHA mutant treated without or with auxin are shown. Parasites (1x$10^7$) treated with 500 µM IAA or carrier solvent (0.1% ethanol, -IAA) were incubated with 0.5 mM [$^{13}$C]-*myo*-inositol (6 h, 37°C), followed by lipidomic analysis (n= 5 assays). No other phospholipid other than PtdIns species (Fig 4e) were labeled with $^{13}$C moiety. PtdIns levels shown here depict the sum of both [$^{12}$C]-PtdIns and [$^{13}$C]-PtdIns. Note that a minor increase in PtdThr, though statistically significant, may have little biological relevance in free tachyzoites.

## Supplementary Figure 3

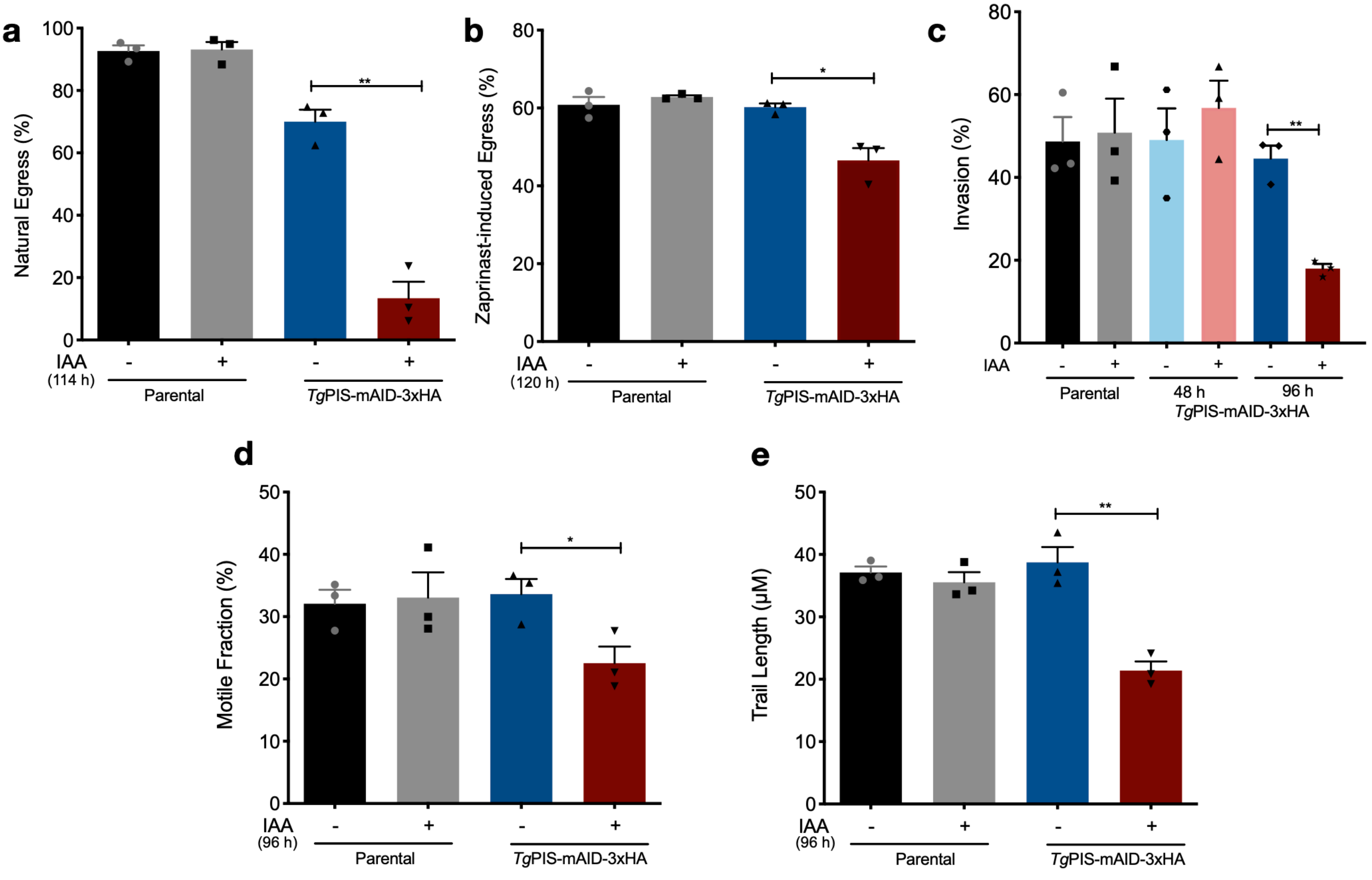


***Tg*PIS depletion impairs the egress, invasion and motility of tachyzoites. (a-b)** Egress efficiency of the indicated parasites following treatment with IAA (500 µM) or 0.1% ethanol (solvent) in the absence (*a*) or presence of zaprinast (*b*). The latter compound inhibits phosphodiesterases, and consequently induces premature egress, presumably by activating cGMP signaling within tachyzoites. Parasites were precultured in the presence of IAA or carrier solvent for 48 h prior to setting up egress assays. Parasitized cultures (control or auxin-treated; 24 h infection) were treated with 500 µM zaprinast for 30 min to measure drug-induced egress. Natural egress (*panel a*) shown here was scored 64 h post-infection (normalized to total number of vacuoles observed 40 h post-infection). Intracellular parasites were stained red by α-*Tg*GAP45 antibody, while extracellular (egressed) ones appeared two-colored (red and green), stained with both α-*Tg*GAP45 and α-*Tg*SAG1 antibodies. The percentage of ruptured vacuoles was determined by numerating 400-500 vacuoles for each strain (n= 3 assays). Note that a severe defect in natural egress of the PIS mutant is likely due to slower replication (see Fig 5c), while a modestly impaired zaprinast-induced egress may be due to defect in the gliding motility (refer to *panel d-e*). **(c)** Invasion rates of the specified tachyzoite strains. 1000-1200 parasites for each strain were scored to calculate the invasion efficiency (means with S.E.; n= 3 assays). **(d-e)** The motile fraction and trail lengths of the *Tg*PIS-mAID-3xHA mutant and parental strain. Samples were stained with α-*Tg*SAG1 antibody. A total of 600 parasites were scored for the motile fraction and 100 trail lengths were measured using ImageJ software (n= 3 assays). For the motility and invasion assays (*panel c-e*), parasites were precultured with 500 µM IAA or 0.1% ethanol for 96 h. Statistical significance was measured by comparing the auxin and ethanol-treated samples (**p*≤0.05; ***p*≤0.01; ****p*≤0.001).

# Supplementary Figure 4

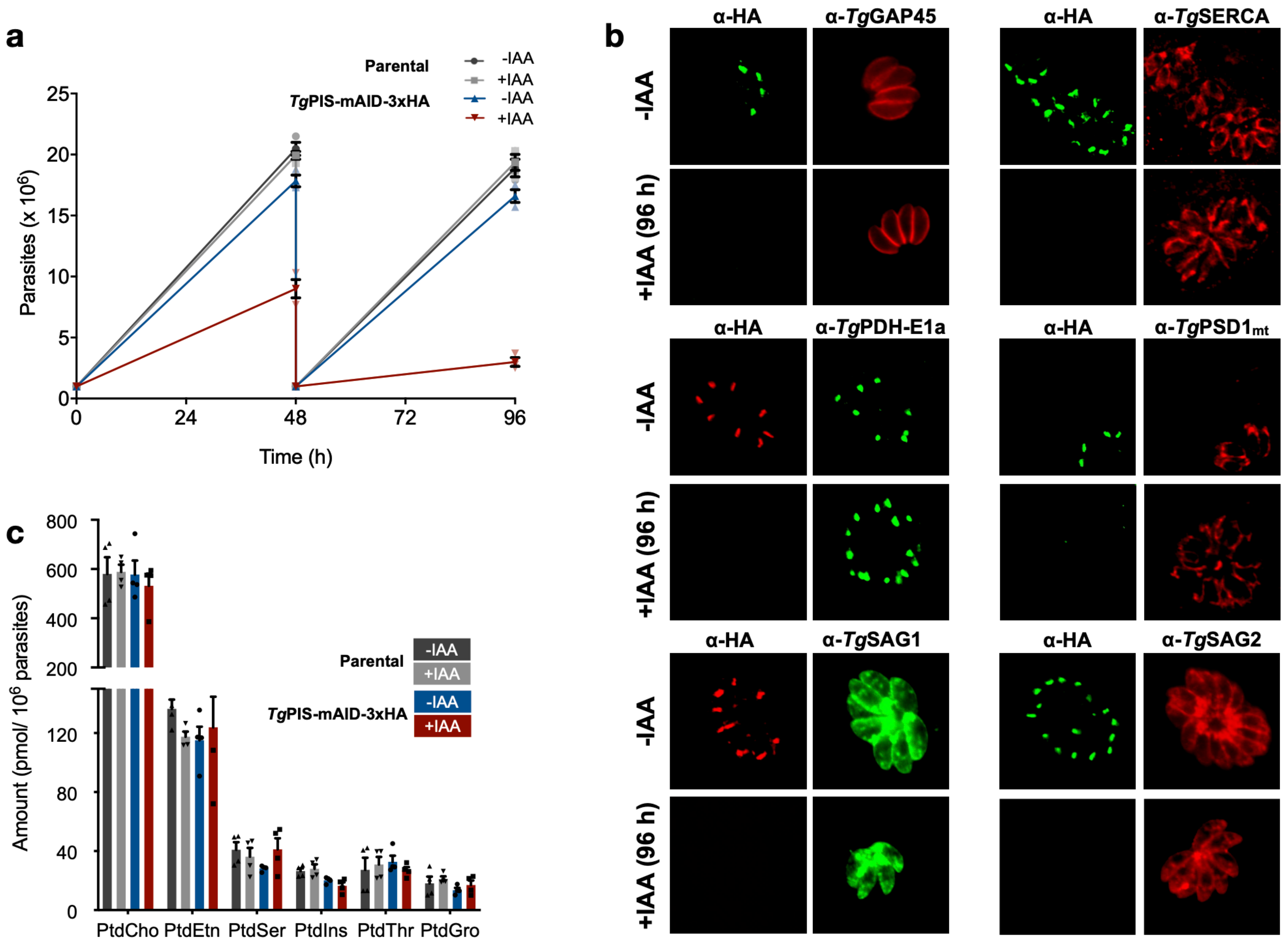


**The parasite yield, organelle morphology and lipid contents after knockdown of PIS. (a)** Yield assay of the parental and *Tg*PIS-mAID-3xHA strains. Fresh tachyzoites (1x$10^6$) of each strain were propagated in confluent HFF cells (MOI, 1) for two passages in the absence or presence of 500 µM IAA or 0.1% ethanol, and the parasite yield was calculated after each passage (n = 3 assays; mean ± S.E.). **(b)** Immunostaining of major organelles in the *Tg*PIS mutant without and with exposure to auxin. The auxin-treated (+IAA) samples were cultured with 500 µM IAA for 72 h prior to immunostaining of the plasma membrane (*Tg*SAG1, *Tg*SAG2), IMC (*Tg*GAP45), apicoplast (*Tg*PDH-E1a), endoplasmic reticulum (*Tg*SERCA) and mitochondrion (*Tg*$PSD1_{mt}$). The α-HA antibody was used to monitor the depletion of *Tg*PIS-3xHA-mAID protein after auxin exposure. **(c)** Amount of individual phospholipid classes in the parental or *Tg*PIS-mAID-3xHA strains, pre-cultured with 500 µM IAA (+IAA) or 0.1% ethanol (-IAA) for two passages (96 h). The sample collection for lipidomics was based on the yield assay (*panel a*) and immunostaining of organelles (*panel b*). The lipid amounts represent the mean ± S.E. (n = 4 assays; *$p \leq 0.05$; **$p \leq 0.01$; ***$p \leq 0.001$).

# Supplementary Figure 5

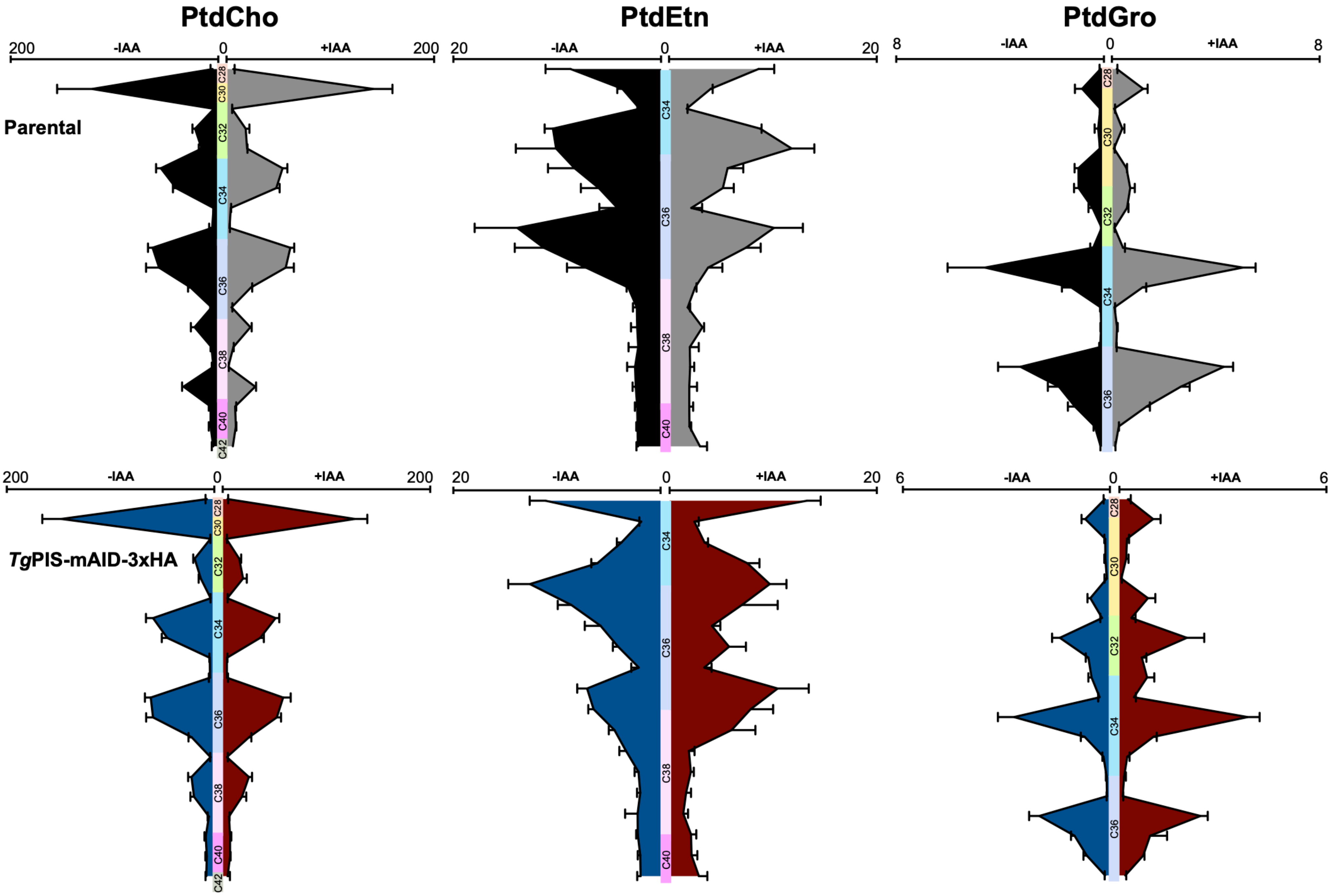


**Downregulation of *Tg*PIS does not affect PtdCho, PtdEtn and PtdGro.** Lipids from the *Tg*PIS-mAID-3xHA (mutant) and RHΔ*ku80*Δ*hxgprt*-TIR1 (parental) strains were analyzed by lipidomic analysis. For each shown lipid class, the amount of all major species (amounting to >90% of total lipid) was plotted (pmol/million parasites) as violin-like plots. Species are ordered with increasing acyl chain length from the top to bottom of the graph, and samples with (+) or without (-) IAA treatments are colored differently. Changes in the graph contour reflects the overall alteration in a lipid. Statistical significance was measured for each lipid species by comparing the auxin-treated and control samples (n= 4 assays; means with S.E.). None of the species qualified the significance test. Note that, although species level changes in PtdGro were not apparent due to minor abundance, its ECN profile indicated a clear increase in the ECN density upon auxin exposure (see Supplementary Figure 6). For the underlying data, refer to Supplementary Data 2.

## Supplementary Figure 6

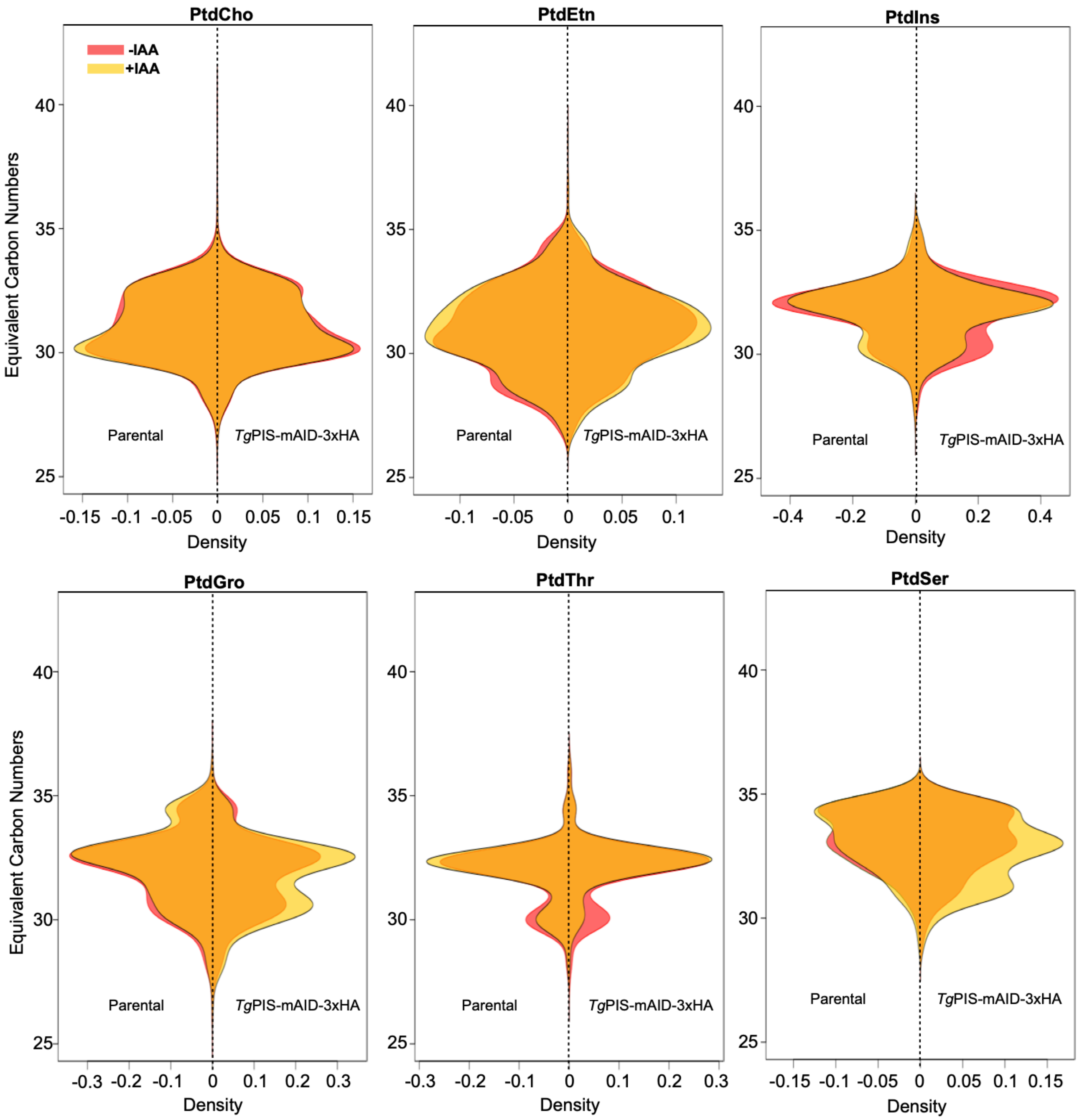


**Knockdown of *Tg*PIS alters the ECN profile of anionic phospholipids in tachyzoites.** Violin plots for the major phospholipid classes based on the acyl chain length and degree of unsaturation. Equivalent carbon number (ECN) density was calculated for all detectable species of each phospholipid, and plotted for the parental strain (*left*) and *Tg*PIS-mAID-3xHA strain (*right*), as shown. In each panel, an overlay of the ECN density is displayed in the presence (yellow) or absence (red) of IAA. Note that the treatment of IAA in both strains has little effect on the ECN profiles of PtdCho and PtdEtn. In contrast, repression of PtdIns synthase resulted in a notable decrease of species with a low ECN in PtdIns and PtdThr (increased red area), while a significant increase in PtdSer and PtdGro (increased yellow area) was detected in the *Tg*PIS-mAID-3xHA strain.

# Supplementary Table 1

| Enzymes | | CDP-alcohol-phosphotransferase motif ($DX_2DGX_2ARX_{8/9}GX_3DX_3D$) | | GenBank No. |
|---|---|---|---|---|
| Phosphatidylinositol Synthase (PIS) | Apicomplexans | ***Tg*PIS** | ..DAVDGAAARRL-GQVSIVGACLDQVVDR.. | KX017549 |
| | | *Nc*PIS | ..DAVDGEAARRL-GQVSVVGACLDQVVDR.. | XP_003879813 |
| | | *Ef*PIS | ..DSVDGFAARSL-KQASVFGACLDQLTDR.. | KX785375 |
| | | *Pf*PIS | ..DALDGWTARKF-NQTSVFGQILDQITDR.. | XP_002809014 |
| | Kinetoplastids | *Tb*PIS | ..DAVDGMVARRF-GQCTQFGAILDMLTDR.. | CAG29793 |
| | | *Tc*PIS | ..DAADGMVARLM-DQCSHFGAIFDMLTDR.. | XP_811459 |
| | | *Lm*PIS | ..DAADGMAARAL-DQCSNFGAILDMLTDR.. | XP_001684255 |
| | Algae | *Cm*PIS | ..DAADGYAARLL-GQSSAFGAVLDMLTDR.. | BAM80990 |
| | | *Cr*PIS | ..DELDGRFARMF-NQTSTFGAVLDMVTDR.. | EDP06395 |
| | | *Ot*PIS | ..DAIDGACARAF-EQSSTFGAALDMITDR.. | CAL56685 |
| | Plants | *At*PIS | ..DAVDGWVARRF-NQVSTFGAVLDMVTDR.. | Q8LBA6 |
| | | *Os*PIS | ..DGLDGWFARKF-NQASTFGAVLDMVTDR.. | CAC37011 |
| | | *Zm*PIS | ..DGVDGWFARKF-NQASTFGAVLDMVTDR.. | NP_001105559 |
| | Fungi | *Cg*PIS | ..DALDGTMARKY-NQVSRLGAVLDMVTDR.. | KTB24417 |
| | | *Sc*PIS | ..DALDGTMARKY-NQVSSLGAVLDMVTDR.. | AAA34876 |
| | | *Sp*PIS | ..DAFDGWAARKL-HQATNFGAILDMVTDR.. | Q10153 |
| | Animals | *Dm*PIS | ..DAVDGQAARAF-NQSTRFGAMLDQLTDR.. | AAF48491 |
| | | *Dr*PIS | ..DAFDGHAARAL-NQGTKFGAMLDMLTDR.. | AAT68039 |
| | | *Mm*PIS | ..DAFDGHAARAL-NQGTRFGAMLDMLTDR.. | Q8VDP6 |
| Choline/Ethanolamine Phosphotransferase (CEPT) | Apicomplexans | *Tg*CEPT1 | ..DAVDGKQARRT-NSSTPLGQLFDHGCDS.. | EPR63017 |
| | | *Tg*CEPT2 | ..DAIDGKHARRN-GLSSPLGQLFDHGCDI.. | EPR62697 |
| | | *Tg*CEPT3 | ..DNVDGKQARRL-RQCTAGGDFLDHSSDS.. | EPR59237 |
| | | *Nc*CEPT1 | ..DAVDGKQARRT-NSSTPLGQLFDHGCDS.. | XP_003883203 |
| | | *Nc*CEPT2 | ..DAIDGKHARRN-SLSSPLGQLFDHGCDI.. | XP_003882816 |
| | | *Nc*CEPT3 | ..DNVDGKQARRL-RQCTAGGDFLDHSSDS.. | XP_003880256 |
| | | *Ef*CEPT1 | ..DAVDGKQARRT-NTATPLGQLFDHGCDS.. | KX785376 |
| | | *Ef*CEPT2 | ..DATDGKHARRL-GLSSPLGQLMDHGCDI.. | KX785377 |
| | | *Ef*CEPT3 | ..DNIDGKQARRL-GLCSAGGDFFDHSSDS.. | KX785378 |
| | | *Pf*CEPT | ..DALDGKQARRT-NTSSPLGQLFDHGCDS.. | XP_966266 |
| | | *Cp*CEPT1 | ..DAADGKHARRL-KISSPLGQLLDHGLDS.. | XP_625864 |
| | | *Cp*CEPT2 | ..DNLDGKQARRL-GVSSNSGEFIDHAIDS.. | XP_625646 |
| | Kinetoplastids | *Tb*CEPT | ..DAIDGKQARRT-NTGSPLGELFDHGCDV.. | XP_823114 |
| | | *Tc*CEPT | ..DAVDGKQARRT-QTCGPLGELFDHGCDA.. | EKG04257 |
| | | *Lm*CEPT | ..DAIDGKQARRT-GTGSPLGELFDHGCDA.. | XP_001687178 |
| | Algae | *Gs*CEPT1 | ..DNLDGRQARRT-NSSSPLGHLFDHGCDA.. | EME27119 |
| | | *Gs*CEPT2 | ..DNLDGRQARRT-NSSSPLGHLFDHGCDA.. | EME32110 |
| | | *Ot*CEPT1 | ..DGMDGKQARRT-KSGSPLGEVIDHACDG.. | XP_003080347 |
| | | *Ot*CEPT2 | ..DGIDGKQARRT-KSGSPLGEVVDHGCDA.. | CEF97354 |
| | Plants | *At*CEPT1 | ..DAVDGKQARRT-NSSSPLGELFDHGCDA.. | O82567 |
| | | *At*CEPT2 | ..DAVDGKQARRT-NSSSPLGELFDHGCDA.. | O82568 |
| | | *Zm*CEPT1 | ..DAVDGKQARRT-SSSSPLGELFDHGCDA.. | XP_008649197 |
| | | *Zm*CEPT2 | ..DAVDGKQARRT-NSSSPLGELFDHGCDA.. | AFW69854 |
| | Fungi | *Cg*CEPT1 | ..DGCDGIHARLT-GQSGPLGELFDHSIDA.. | KTB18544 |
| | | *Cg*CEPT2 | ..DACDGMHARRT-GQSSPLGELFDHCIDS.. | KTB25778 |
| | | *Sc*CEPT1 | ..DGCDGVHARRI-NQSGPLGELFDHSIDA.. | P22140 |
| | | *Sc*CEPT2 | ..DACDGMHARRT-GQQGPLGELFDHCIDS.. | AAA63571 |
| | Animals | *Dm*CEPT | ..DGMDGKQARRT-GTSGPLGELFDHGLDS.. | NP_609149 |
| | | *Dr*CEPT | ..DAIDGKQARRT-NSSSPLGELFDHGCDS.. | NP_001103187 |
| | | *Mm*CEPT | ..DAIDGKQARRT-NSCSPLGELFDHGCDS.. | NP_001140162 |
| Phosphatidylserine Synthase (PSS) | Bacteria | *Bs*PSS | ..DFFDGMAARKL-NAVSDMGRELDSFADL.. | BAA07225 |
| | | *Hp*PSS | ..DGLDGRVARLT-NTTSKFGIEFDSLADV.. | AAC45587 |
| | | *Mt*PSS | ..DGLDGRVARIL-DAQSRMGAEIDSLADA.. | GAA44221 |
| | Fungi | *Cg*PSS | ..DFFDGRVARLR-NRSSLMGQELDSLADL.. | KTB22122 |
| | | *Sc*PSS | ..DFLDGRVARLR-NRSSLMGQELDSLADL.. | BAA00121 |
| | | *Sp*PSS | ..DFLDGKVARWR-GKSSLMGQELDSLADL.. | O94584 |
| Phosphatidylglycerol Phosphate Synthase (PGPS) | Bacteria | *Ec*PGPS | ..DWFDGFLARRW-NQSTRFGAFLDPVADK.. | AAA98754 |
| | | *Hp*PGPS | ..DLLDGYIARSY-KAKSRFGEIFDPVADK.. | EIE30130 |
| | Algae | *Cm*PGPS | ..DWLDGYIARRL-NVSSVWGAFLDPVADK.. | BAM80263 |
| | | *Cr*PGPS | ..DYFDGYLARKL-KIATVFGAFLDPVADK.. | EDO97733 |
| | Plants | *At*PGPS1 | ..DWLDGYLARKM-RLGSAFGAFLDPVADK.. | O80952 |
| | | *At*PGPS2 | ..DWLDGYIARKM-RLGSEFGAFLDPVADK.. | Q9M2W3 |
| | | *Os*PGPS | ..DWLDGYIARKM-QLGTPFGAFLDPVADK.. | XP_015630365 |
| Cardiolipin Synthase (CLS) | Algae | *Cm*CLS | ..DVLDGYLARKY-QKVTTLGSILDPVADK.. | BAM81296 |
| | | *Cr*CLS | ..DWLDGWLARRL-GASSVFGSYLDPLADK.. | XP_001699073 |
| | | *Ot*CLS | ..DYLDGFLARRW-KQQTILGSYLDPVADK.. | XP_003082955 |
| | Plants | *At*CLS | ..DWLDGYVARRM-KINSVVGSYLDPLADK.. | Q93YW7 |
| | | *Os*CLS | ..DWLDGFLARKM-GINSVFGSYLDPLADK.. | Q5N9A1 |
| | Fungi | *Cg*CLS | ..DFLDGYIARRY-NMKSDAGTILDPMADK.. | KTB14470 |
| | | *Sc*CLS | ..DFMDGYIARKY-GLKTIAGTILDPLADK.. | NP_010139 |
| | | *Sp*CLS | ..DLVDGYIARKF-DLGSIAGTVLDPLADK.. | CAB16578 |
| | Animals | *Dm*CLS | ..DLLDGQIARRWPSQASKFGSFLDPMADK.. | NP_651418 |
| | | *Dr*CLS | ..DLLDGYIARNWPNQKSALGSALDPLADK.. | NP_998096 |
| | | *Mm*CLS | ..DLLDGFIARNWANQKSALGSALDPLADK.. | AAH48702 |

**Sequence alignment of CDP-alcohol-phosphotransferase motifs from representative proteins across the tree of life identifies the signature residues of PtdIns synthases.** Conserved motifs of indicated sequences were predicted by Simple Modular Architecture Research Tool and then aligned by the CLC workbench. Proteins were chosen based on the CDP-alcohol-phosphotransferase motif shown in the top row. Organism abbreviations: *Tg*, *Toxoplasma gondii*; *Nc*, *Neospora caninum*; *Ef*, *Eimeria falciformis*; *Pb*, *Plasmodium berghei*; *Pf*, *Plasmodium falciparum*; *Tb*, *Trypanosoma brucei*; *Tc*, *Trypanosoma cruzi*; *Lm*, *Leishmania major*; *Cm*, *Cyanidioschyzon merolae*; *Cr*, *Chlamydomonas reinhardtii*; *Ot*, *Ostreococcus tauri*; *At*, *Arabidopsis thaliana*; *Os*, *Oryza sativa*; *Zm*, *Zea mays*; *Cg*, *Candida glabrata*; *Sc*, *Saccharomyces cerevisiae*; *Sp*, *Schizosaccharomyces pombe*; *Dm*, *Drosophila melanogaster*; *Dr*, *Danio rerio*; *Hs*, *Homo sapiens*; *Cp*, *Cryptosporidium parvum*; *Gs*, *Galdieria sulphuraria*; *Bs*, *Bacillus subtilis*; *Hp*, *Helicobacter pylori*; *Mt*, *Mycobacterium tuberculosis*; *Ec*, *Escherichia coli*.

# Supplementary Table 2

| Primer Name<br>(restriction site) | Primer Sequence<br>(restriction site underlined) | Cloning Vector<br>(research objectives) |
|---|---|---|
| **Ectopic expression of *Tg*PIS$^{49-258}$-HA and *Tg*PIS-HA (RHΔ*ku80*-TaTi strain)** | | |
| *Tg*PIS$^{49-258}$-F (*Nco*I)<br>*Tg*PIS-HA-R (*Pac*I) | CTCATCCCATGGTTTTCCTCTACGTGCCAA<br>CTCATCTTAATTAATCAAGCGTAATCTGGAACATCGTATGGGTACGACGAGGGCGCACCAAA | *pTETO7SAG1-UPKO* (Ectopic expression of *Tg*PIS$^{49-258}$-HA) |
| *Tg*PIS-Myc-F (*Nco*I)<br>*Tg*PIS-HA-R (*Pac*I) | CTCATCCCATGGAACAGAAGTTGATTTCCGAAGAAGACCTCGCGGGGACTTCTGCAAGCC<br>CTCATCTTAATTAATCAAGCGTAATCTGGAACATCGTATGGGTACGACGAGGGCGCACCAAA | *pTETO7SAG1-UPKO* (Ectopic expression of Myc-*Tg*PIS-HA) |
| **Functional expression of *Tg*PIS-6xHis and its mutants (*E. coli* M15/pREP4 strain)** | | |
| *Tg*PIS-QE-F (*Bgl*II)<br>*Tg*PIS-QE-R (*Bgl*II) | CTCATCAGATCTATGGCGGGGACTTCTGCAAG<br>CTCATCAGATCTCGACGAGGGCGCACCAAA | *pQE60* (Functional expression of *Tg*PIS-6xHis) |
| *Tg*PIS$^{49-258}$-QE-F (*Bgl*II)<br>*Tg*PIS-QE-R (*Bgl*II) | CTCATCAGATCTATGGTTTTCCTCTACGTGCCAAACA<br>CTCATCAGATCTCGACGAGGGCGCACCAAA | *pQE60* (Functional expression of *Tg*PIS$^{49-258}$-6xHis) |
| *Tg*PIS$^{91D-A}$-F<br>*Tg*PIS$^{91D-A}$-R | GCCGCAGTCGATGGCG<br>CAGGCATTGCGATGTAACA | *pQE60-Tg*PIS (Functional expression of *Tg*PIS$^{91D-A}$) |
| *Tg*PIS$^{94D-A}$-F<br>*Tg*PIS$^{94D-A}$-R | GCTGGCGCTGCCGC<br>GACTGCGTCCAGGCATTG | *pQE60-Tg*PIS (Functional expression of *Tg*PIS$^{94D-A}$) |
| *Tg*PIS$^{103Q-G}$-F<br>*Tg*PIS$^{103Q-G}$-R | GGAGTTTCCATTGTCGGCG<br>GCCCAGACGGCGTG | *pQE60-Tg*PIS (Functional expression of *Tg*PIS$^{103Q-G}$) |
| *Tg*PIS$^{112D-A/113Q-G}$-F<br>*Tg*PIS$^{112D-A/113Q-G}$-R | GCCGGAGTTGTCGACCGG<br>GAGACAGGCGCCGACA | *pQE60-Tg*PIS (Functional expression of *Tg*PIS$^{112D-A/113Q-G}$) |
| *Tg*PIS$^{116D-A/117R-G}$-F<br>*Tg*PIS$^{116D-A/117R-G}$-R | GCCGGGCTTTCAACATGTCT<br>GACAACTTGGTCGAGACAGG | *pQE60-Tg*PIS (Functional expression of *Tg*PIS$^{116D-A/117R-G}$) |
| **Making the *Tg*PIS-loxP mutant (RHΔ*ku80*Δ*hxgprt* strain)** | | |
| *Tg*PIS-Cre-F (*EcoR*I)<br>*Tg*PIS-HA-R (*Pac*I) | CTCATCGAATTCCGACAAAATGGCGGGGACTTCTGC<br>CTCATCTTAATTAATCAAGCGTAATCTGGAACATCGTATGGGTACGACGAGGGCGCACCAAA | *pG140* (Homologous expression of *Tg*PIS-HA) |
| *Tg*PIS-5'UTR-Cre-F (*Apa*I)<br>*Tg*PIS-5'UTR-Cre-R (*EcoR*I) | CTCATCGGGCCCATGGCCAACAGCTCAGCT<br>CTCATCGAATTCTATAACTTCGTATAATGTATGCTATACGAAGTTATGAGCCGCTTTTCTTTTTGC<br>→ loxP-site loxP-site ← | *pG140* (Homologous recombination at 5' end) |
| *Tg*PIS-3'UTR-Cre-F (*Sac*I)<br>*Tg*PIS-3'UTR-Cre-R (*Sac*I) | CTCATCGAGCTCGGTCGCTTTTCTCGTGAAGA<br>CTCATCGAGCTCTTGTGCGAACTAGACGTATGACTT | *pG140* (Homologous recombination at 3' end) |
| *Tg*PIS-loxP-5'Scr-F<br>*Tg*PIS-loxP-5'Scr-R | CTCGATATGGGGAACCCAA<br>CAGATGAACTTCAGGGTCAGC | *pDrive* (*Tg*PIS 5'Scr PCR product for verification) |
| *Tg*PIS-loxP-3'Scr-F<br>*Tg*PIS-loxP-3'Scr-R | CTACGACTTCAACGAGATGTTCC<br>GTCCAGTGTTCAGGACATTGAGT | *pDrive* (*Tg*PIS 3'Scr PCR product for verification) |
| *Tg*PIS-loxP-IScr-F<br>*Tg*PIS-loxP-IScr-R | GTCAGATTCTGACCTTTCTCATGA<br>GAACGGTAGTAGACGGAGAGAATG | *pDrive* (*Tg*PIS internal Scr PCR product for verification) |
| **Making the *Tg*PIS-mAID-3xHA mutants(RHΔ*ku80Δhxgprt*-TIR1)** | | |
| pU6-TgPIS-3HA-mAID-F<br>pU6-TgPIS-3HA-mAID-R | AAGTTGAGGCAAGACTGTTTTTCAGG<br>AAAACCTGAAAAACAGTCTTGCCTCA | *pU6-Universal* (Cas9-based DNA cleavage at *Tg*PIS-3'UTR) |
| TgPIS-mAID-3HA-F<br>TgPIS-mAID-3HA-R | GCGCTTGGTTGCGTGCGACATTTTTGGTGCGCCCTCGTCGGCTAGCAAGGGCTCGGGCTCGA CCCAGC<br>GAGAACTTGTGGCTGCTCTCAGGGTAGGTCTCCCCAGAGGATAGGGCGAATTGGAGCTCC | pLinker-3xHA-mAID(Amplify PCR-based templates to assist homology direct repair at *Tg*PIS-3'UTR) |
| TgPIS-mAID-Scr-F<br>TgPIS-mAID-Scr-R | CTGACGAATCTCCTCCAAGGAC<br>ATAGGGCGAATTGGAGCTCC | *pDrive*(Sequencing of *Tg*PIS-mAID-Scr product) |

**Oligonucleotides, plasmid constructs and the parasite strains used in this study.**

# Uncropped Gel/Blot Images

**Figure 2b**

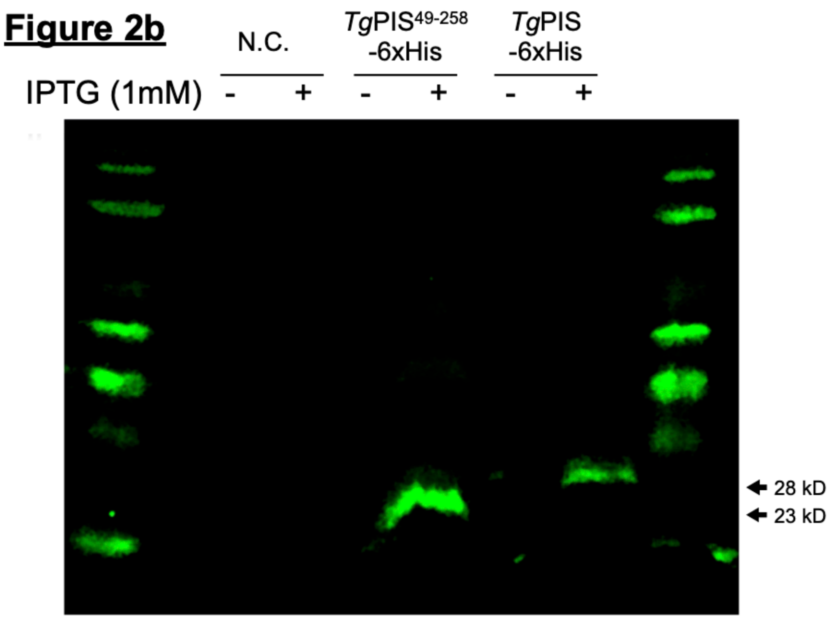


**Figure 4b**

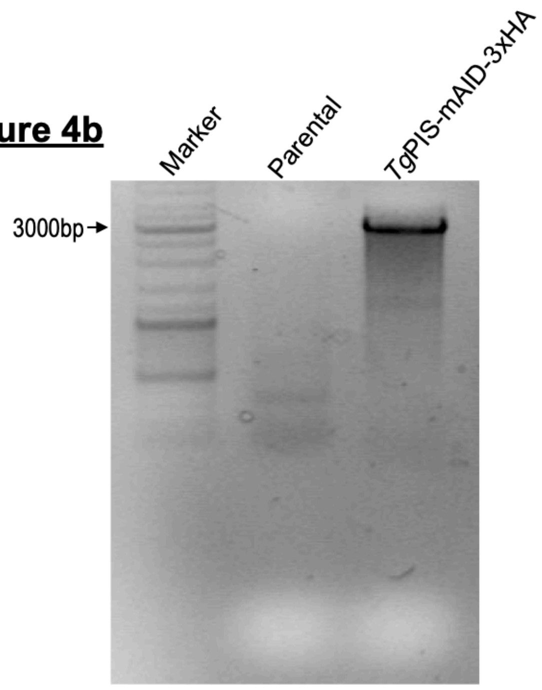


**Figure 3b**

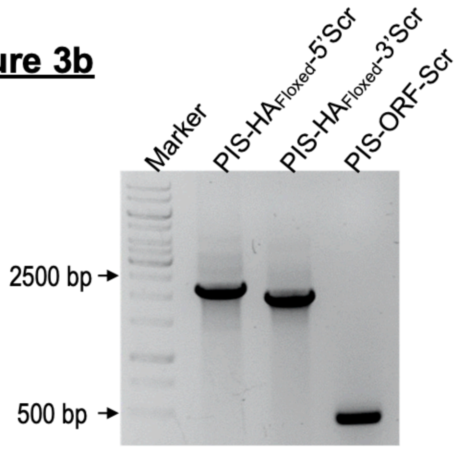


**Figure 4d**

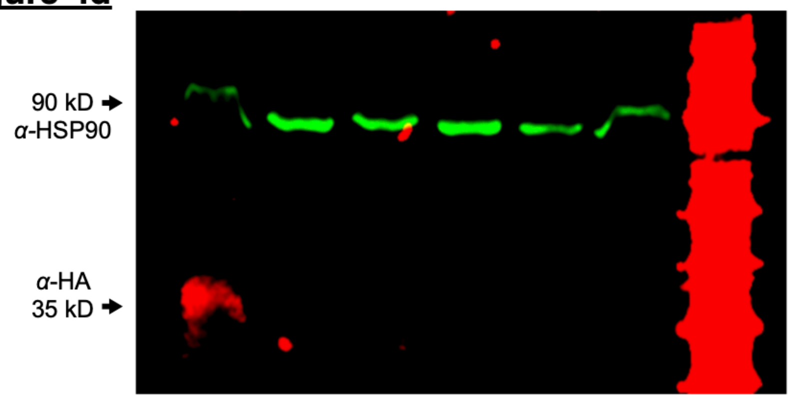

Supplement: Supplementary file 1 — Supplementary Information [file 42003_2020_1480_MOESM1_ESM.pdf]
