# Supplementary material for: Phosphatidylinositol synthesis, its selective salvage, and inter-regulation of anionic phospholipids in Toxoplasma gondii
Source: Commun Biol. 2020 Dec 10;3:750. doi: 10.1038/s42003-020-01480-5 (PMC7728818; doi:10.1038/s42003-020-01480-5)
Supplement: Supplementary file 2 — Description of Additional Supplementary Files [file 42003_2020_1480_MOESM2_ESM.pdf]

Description of Additional Supplementary Files

Title: Supplementary Data 1

Description: The numerical values used to generate graphs presented in our work

Title: Supplementary Data 2

Description: Lipidomic data underlying the lipid species shown in Figure 6 and Figure S5

Title: Supplementary Data 3

Description: Lipidomic data underlying the volcano plots (Figure 7
