# Supplementary material for: Phosphatidylinositol synthesis, its selective salvage, and inter-regulation of anionic phospholipids in Toxoplasma gondii
Source: Commun Biol. 2020 Dec 10;3:750. doi: 10.1038/s42003-020-01480-5 (PMC7728818; doi:10.1038/s42003-020-01480-5)
Supplement: Supplementary file 6 — Reporting Summary [file 42003_2020_1480_MOESM6_ESM.pdf]

nature research

Corresponding author(s): Nishith Gupta
Last updated by author(s): Sep 1, 2020

Reporting Summary

Nature Research wishes to improve the reproducibility of the work that we publish. This form provides structure for consistency and transparency in reporting. For further information on Nature Research policies, see our Editorial Policies and the Editorial Policy Checklist.

Statistics

For all statistical analyses, confirm that the following items are present in the figure legend, table legend, main text, or Methods section.

n/a	Confirmed	
☐	☒	The exact sample size (n) for each experimental group/condition, given as a discrete number and unit of measurement
☐	☒	A statement on whether measurements were taken from distinct samples or whether the same sample was measured repeatedly
☐	☒	The statistical test(s) used AND whether they are one- or two-sided<br>*Only common tests should be described solely by name; describe more complex techniques in the Methods section.*
☐	☒	A description of all covariates tested
☐	☒	A description of any assumptions or corrections, such as tests of normality and adjustment for multiple comparisons
☐	☒	A full description of the statistical parameters including central tendency (e.g. means) or other basic estimates (e.g. regression coefficient) AND variation (e.g. standard deviation) or associated estimates of uncertainty (e.g. confidence intervals)
☒	☐	For null hypothesis testing, the test statistic (e.g. F, t, r) with confidence intervals, effect sizes, degrees of freedom and P value noted<br>*Give P values as exact values whenever suitable.*
☒	☐	For Bayesian analysis, information on the choice of priors and Markov chain Monte Carlo settings
☒	☐	For hierarchical and complex designs, identification of the appropriate level for tests and full reporting of outcomes
☒	☐	Estimates of effect sizes (e.g. Cohen's d, Pearson's r), indicating how they were calculated

Our web collection on statistics for biologists contains articles on many of the points above.

Software and code

Policy information about availability of computer code

Data collection	No software was used.
Data analysis	Prism 8

For manuscripts utilizing custom algorithms or software that are central to the research but not yet described in published literature, software must be made available to editors and reviewers. We strongly encourage code deposition in a community repository (e.g. GitHub). See the Nature Research guidelines for submitting code & software for further information.

Data

Policy information about availability of data

All manuscripts must include a data availability statement. This statement should provide the following information, where applicable:
- Accession codes, unique identifiers, or web links for publicly available datasets
- A list of figures that have associated raw data
- A description of any restrictions on data availability

The dataset generated are presented in their entirety in the manuscript.

Field-specific reporting

Life sciences study design

All studies must disclose on these points even when the disclosure is negative.

Sample size	All data in the manuscript consist of at least three biological replicates.
Data exclusions	No data was excluded in the study.
Replication	All data and experiments are reproducible, and repeated at least 3 times.
Randomization	The samples underlying this study do not require randomization.
Blinding	No blinding is required.

Reporting for specific materials, systems and methods

We require information from authors about some types of materials, experimental systems and methods used in many studies. Here, indicate whether each material, system or method listed is relevant to your study. If you are not sure if a list item applies to your research, read the appropriate section before selecting a response.

Materials & experimental systems

n/a	Involved in the study	
☐	☒	Antibodies
☐	☒	Eukaryotic cell lines
☒	☐	Palaeontology and archaeology
☒	☐	Animals and other organisms
☒	☐	Human research participants
☒	☐	Clinical data
☒	☐	Dual use research of concern

Methods

n/a	Involved in the study	
☒	☐	ChIP-seq
☒	☐	Flow cytometry
☒	☐	MRI-based neuroimaging

Antibodies

Antibodies used	Antibodies recognizing the TgHSP90, TgGAP45, TgPDH-E1a, TgSAG2 and TgIMC3 proteins were bestowed by Sergio Angel (IIB-INTECH, Argentina), Dominique Soldati-Favre (University of Geneva, Switzerland), Bang Shen (Huazhong Agricultural University, China), Honglin Jia (Harbin Veterinary Research Institute, China) and Marc-Jan Gubbels (Boston College, MA, USA), respectively. Anti-TgSERCA and anti-TgPSD1mt antisera were obtained from FriendBio Bioscience and Technology (China). Other primary antibodies recognizing the TgSAG1 protein and engineered epitopes (HA and Ty1) were obtained from ThermoFisher Scientific and Sigma-Aldrich. Corresponding secondary antibodies (Alexa488, Alexa594) were procured from Life Technologies (Germany).
Validation	All antibodies are published and acknowledged appropriately in the manuscript.

Eukaryotic cell lines

Policy information about cell lines

Cell line source(s)	Human Foreskin Fibroblast (HFF) cell lines.
Authentication	None of the cell lines used here was authenticated.
Mycoplasma contamination	No test for mycoplasma contamination were performed.
Commonly misidentified lines (See ICLAC register)	n/a
